# Nipah virus induces two inclusion body populations: Identification of novel inclusions at the plasma membrane

**DOI:** 10.1371/journal.ppat.1007733

**Published:** 2019-04-29

**Authors:** Marc Ringel, Anja Heiner, Laura Behner, Sandro Halwe, Lucie Sauerhering, Nico Becker, Erik Dietzel, Bevan Sawatsky, Larissa Kolesnikova, Andrea Maisner

**Affiliations:** 1 Institute of Virology, Philipps University Marburg, Marburg, Germany; 2 Division of Veterinary Medicine, Paul-Ehrlich-Institut, Langen, Germany; University of Kentucky, UNITED STATES

## Abstract

Formation of cytoplasmic inclusion bodies (IBs) is a hallmark of infections with non-segmented negative-strand RNA viruses (order *Mononegavirales*). We show here that Nipah virus (NiV), a bat-derived highly pathogenic member of the *Paramyxoviridae* family, differs from mononegaviruses of the *Rhabdo-*, *Filo-* and *Pneumoviridae* families by forming two types of IBs with distinct localizations, formation kinetics, and protein compositions. IBs in the perinuclear region form rapidly upon expression of the nucleocapsid proteins. These IB_peri_ are highly mobile and associate with the aggresome marker y-tubulin. IB_peri_ can recruit unrelated overexpressed cytosolic proteins but do not contain the viral matrix (M) protein. Additionally, NiV forms an as yet undescribed IB population at the plasma membrane (IB^PM^) that is y-tubulin-negative but contains the M protein. Infection studies with recombinant NiV revealed that IB^PM^ require the M protein for their formation, and most likely represent sites of NiV assembly and budding. The identification of this novel type of plasma membrane-associated IBs not only provides new insights into NiV biology and may open new avenues to develop novel antiviral approaches to treat these highly pathogenic viruses, it also provides a basis for a more detailed characterization of IBs and their role in virus assembly and replication in infections with other *Mononegavirale*s.

## Author summary

Inclusion bodies (IBs) induced by non-segmented negative-strand RNA viruses (*Mononegavirales*) are described as mobile cytosolic compartments that concentrate viral proteins and represent the main viral replication sites in infected cells. This general concept is mainly based on studies with mononegaviruses from the *Rhabdo-*, *Filo-* and *Pneumoviridae* families. IBs induced by members of the *Paramyxoviridae* family are much less well characterized, and this study provides evidence that paramyxoviral IBs may have different compositions and functions. The main finding of this study is that Nipah virus (NiV), a highly pathogenic member of the genus *Henipavirus* in the family *Paramyxoviridae*, forms a novel type of IB whose formation at plasma membrane assembly sites depends on the viral matrix protein, and suggests a role for IBs not yet described for other *Mononegavirales*. This discovery clearly extents the current concept of IB functions and illustrates the need to further investigate IBs formed by other paramyxoviruses.

## Introduction

Nipah virus (NiV) and the closely related Hendra virus (HeV) are members of the Henipavirus genus in the family *Paramyxoviridae* (order *Mononegavirales*) [1]. NiV initially emerged in peninsular Malaysia and Singapore in 1998/99 associated with a large outbreak of respiratory disease in pigs and encephalitis among pig farm and abattoir workers [2]. The Bangladesh strain of NiV has caused sporadic outbreaks of highly fatal encephalitis since then in Bangladesh and eastern India, as well as the recent outbreak in Kerala state in southwestern India [3]. Due to its high lethality, work on infectious NiV is confined to high-containment biosafety level 4 (BSL-4) laboratories.

NiV has a negative-stranded RNA genome which encodes six structural proteins. The genomic RNA together with the nucleoprotein (N), the phosphoprotein (P) and viral polymerase (L) form the nucleocapsid (NC) which is surrounded by a lipid envelope that is derived from the plasma membrane during the budding process. Both NiV surface glycoproteins, the receptor-binding (G) and the fusion (F) protein, are incorporated into the viral envelope. The NiV glycoproteins mediate pH-independent fusion events with the host cell membrane either during virus entry or during direct virus spread via cell-cell fusion [4]. The NiV matrix (M) protein is associated with the inner leaflet of the viral membrane and acts as a bridge between the NC and surface glycoproteins during assembly. Due to its interactions with other viral proteins, cellular proteins and lipids, the M protein plays a central role in virus particle assembly [5–9]. Though NiV replicates in the cytoplasm, the M protein transits through the nucleus by virtue of its nuclear import and export signals, before ultimately coordinating the assembly of NiV particles at the plasma membrane [10–15]. We and others have recently characterized the functional role of the NiV M protein in more detail by generating an M-deficient recombinant NiVΔM [16, 17]. Due to the drastic assembly defect, spread of NiVΔM in cell cultures occurs almost exclusively via cell-cell fusion that likely allows cytoplasmic viral nucleocapsids to directly spread to adjacent uninfected cells in the absence of virus budding [18]. While budding of correctly assembled, infectious virus particles was greatly reduced, viral RNA synthesis and viral protein synthesis in infected cells was not markedly different compared to wildtype infection [16]. This indicated that the NiV M protein is essential for assembly and budding but does not play a central role in virus replication and transcription processes.

Inclusion body (IB) formation is a hallmark of infections by members of the *Mononegavirales*. Due to the concentration of viral components such as viral RNA and nucleocapsid proteins at these sites, IBs are thought to be sites of viral replication and transcription. Indeed, prior studies have demonstrated that *de novo* RNA synthesis occurs in IBs formed by different viruses, such as respiratory syncytial virus (RSV), human metapneumovirus (HPMV), Ebola virus (EBOV), Marburg virus (MARV), rabies virus (RABV), and vesicular stomatitis virus (VSV) [19–25]. After viral RNA synthesis in IBs it is believed that viral mRNAs are exported to and translated by cytoplasmic ribosomes. Genomic RNA is packaged into viral NCs that are then transported to the plasma membrane where virus assembly and budding occurs.

As with other members of the *Paramyxoviridae* family, the cytopathic effect in NiV-infected cells involves the formation of multinucleated syncytia due to pH-independent glycoprotein mediated cell-cell fusion between adjacent cells. Earlier ultrastructural analyses showed the accumulation of herringbone-like NiV NCs closely aligned along the plasma membrane, in preparation for packaging into new virus particles [26–28]. Viral NC inclusions were also formed in the cytoplasm. While cytoplasmic inclusions by members of the *Mononegavirales* are often presumed to be viral factories in which replication and transcription occurs, some early studies of NiV indicated that the function of cytoplasmic NiV IBs may be somewhat different. Although IBs containing NiV NCs were strongly positive for NiV proteins, viral RNA was mostly associated with cytoplasmic networks of membrane-like reticular structures [28]. An earlier study also demonstrated that NiV NCs form aggregates both *in vitro* and *in vivo* that were located predominantly at the periphery of syncytia rather than closer to the center of infected cells [27]. Taken together, these lines of evidence point to the existence of IBs in NiV-infected cells with some exceptional properties, and perhaps even to different functional roles during infection. We characterized the requirements of NiV IB formation, as well as their subcellular localization and protein composition, using both infectious virus and plasmid-based transfections to answer the question of how these IBs differ compared to other members of the *Mononegavirales* order. We have identified two IB subpopulations, IB_peri_ and IB^PM^, that differed in their intracellular localizations, their formation kinetics and their protein compositions. IB_peri_ were formed as soon as NiV nucleocapsid proteins were expressed and were spread throughout the cytoplasm, mostly in perinuclear regions. These IB_peri_ did not contain the NiV M protein, but did contain the cellular aggresome marker y-tubulin (M-negative, y-tubulin-positive). IB^PM^ were found in close association with the plasma membrane and were formed later because they require a functional expression of the NiV M protein. In contrast to IB_peri_, IB^PM^ clearly colocalized with the M protein but did not concentrate y-tubulin (M-positive, y-tubulin-negative).

## Results

### NiV infection induces the formation of perinuclear and peripheral inclusion bodies containing NC-like structures

**Fig 1:**
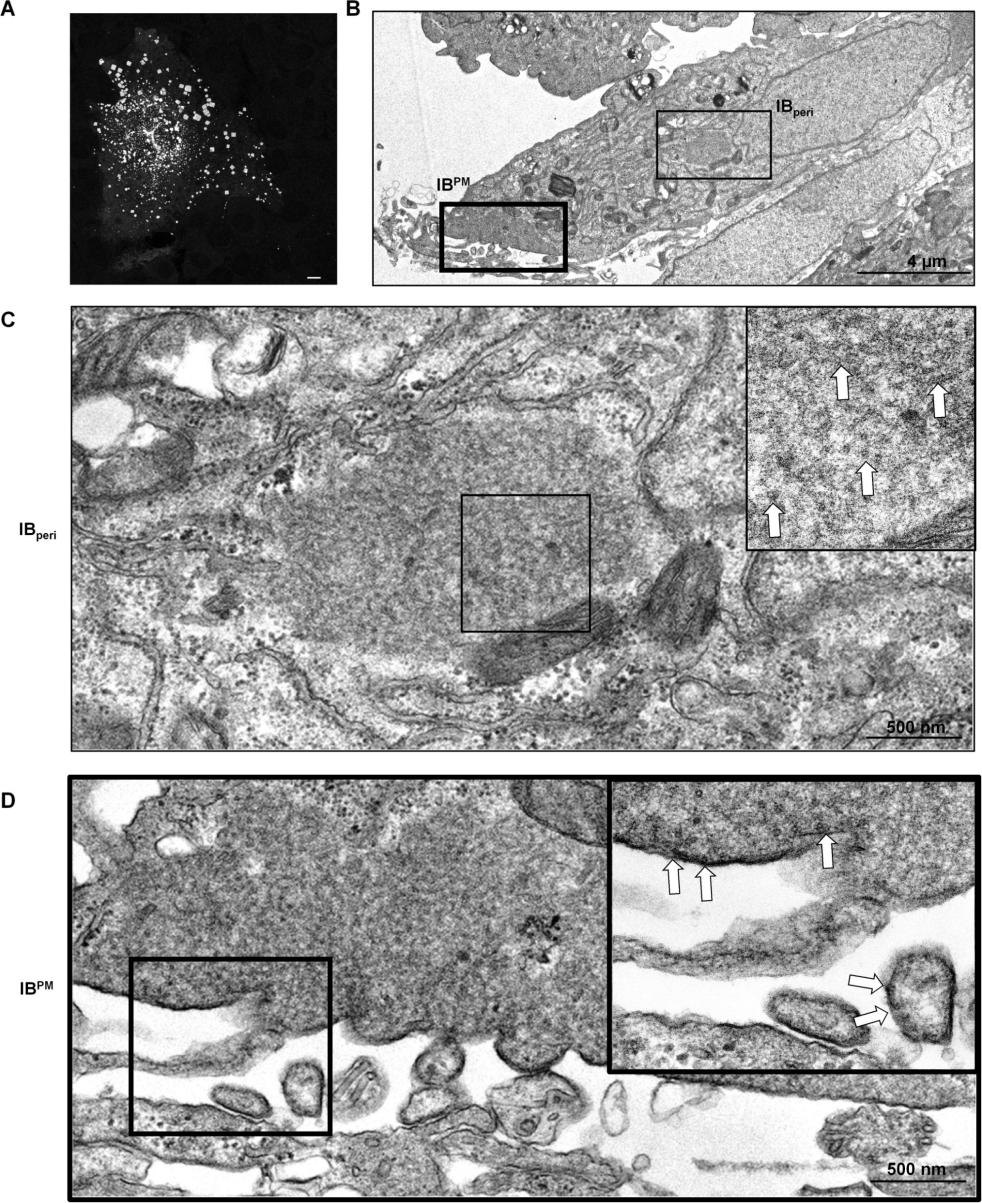
Inclusion bodies (IBs) in NiV-infected cells. **(A)** Vero76 cells were infected with wildtype NiV at a multiplicity of infection (MOI) of 0.05. At 24 h p.i. cells were fixed with 4% PFA for 48 h and permeabilized with Triton X-100. For staining of NiV IBs in syncytia, permeabilized cells were incubated with NiV N-specific antiserum. Scale bar, 10 μm. **(B-D)** For ultrastructural analysis, cells were infected with NiV at a MOI of 2. Infected cells were fixed and processed for transmission electron microscopy at 24 h p.i.. **(B)** Low magnification overview of a NiV-positive cell containing a perinuclear IB (IB_peri_) and an IB associated with the plasma membrane (IB^PM^). **(C, D)** Enlarged views of IB_peri_ (C) and IB^PM^ (D) marked in the overview in (B) are shown. Boxed areas are shown at higher magnifications. White arrows indicate mostly cross-sectioned viral nucleocapsids within IB_peri,_ or aligned at the plasma membrane in IB^PM^.

Inclusions formed by members of the *Mononegavirales* are found largely in the perinuclear region or are randomly distributed in infected cells. A close association between IBs and the plasma membrane has not been specifically described thus far. Prior studies, however, have revealed that NiV inclusions containing the NCs were predominantly located in the periphery of syncytia rather than around the nucleus or randomly distributed throughout the cytoplasm [27]. This localization at the plasma membrane suggests a hitherto unknown functional role for these inclusions. To test this idea, we infected Vero76 cells with NiV at a multiplicity of infection (MOI) of 0.05. Cytoplasmic IBs contain large amounts of the viral N and P proteins, so IB formation was analyzed by immunostaining with an anti-NiV N guinea pig serum. At 24 h post infection (p.i.), multiple IBs were formed within NiV-induced syncytia (Fig 1A), and these IBs were found in the syncytium periphery, and also partially located close to nuclei. Subsequent ultrastructural analysis of thin sections of infected cells supported the notion of two populations of IBs in different subcellular locations. NiV-infected cells contained IBs in the perinuclear region (designated IB_peri_) as well as IBs in close association with the plasma membrane (designated IB^PM^) (Fig 1B). IB^PM^ often cover large areas of the plasma membranes and display different morphologies ranging from thin layers underneath the plasma membrane to almost square-shaped structures (S1 Fig). Typical helical paramyxovirus nucleocapsid-like structures were found in both IB populations (Fig 1C and 1D). Though NCs are often difficult to identify within normal cellular structures, we also detected them in the cytoplasm outside of IBs (S2 Fig). NCs were also found to be aligned beneath the plasma membrane in IB^PM^, and were closely associated with budding events (Fig 1D, insert), suggesting that IB^PM^ may provide a platform for viral assembly at the plasma membrane.

### NiV lacking the M gene does not form inclusion bodies at the plasma membrane (IB^PM^)

**Fig 2:**
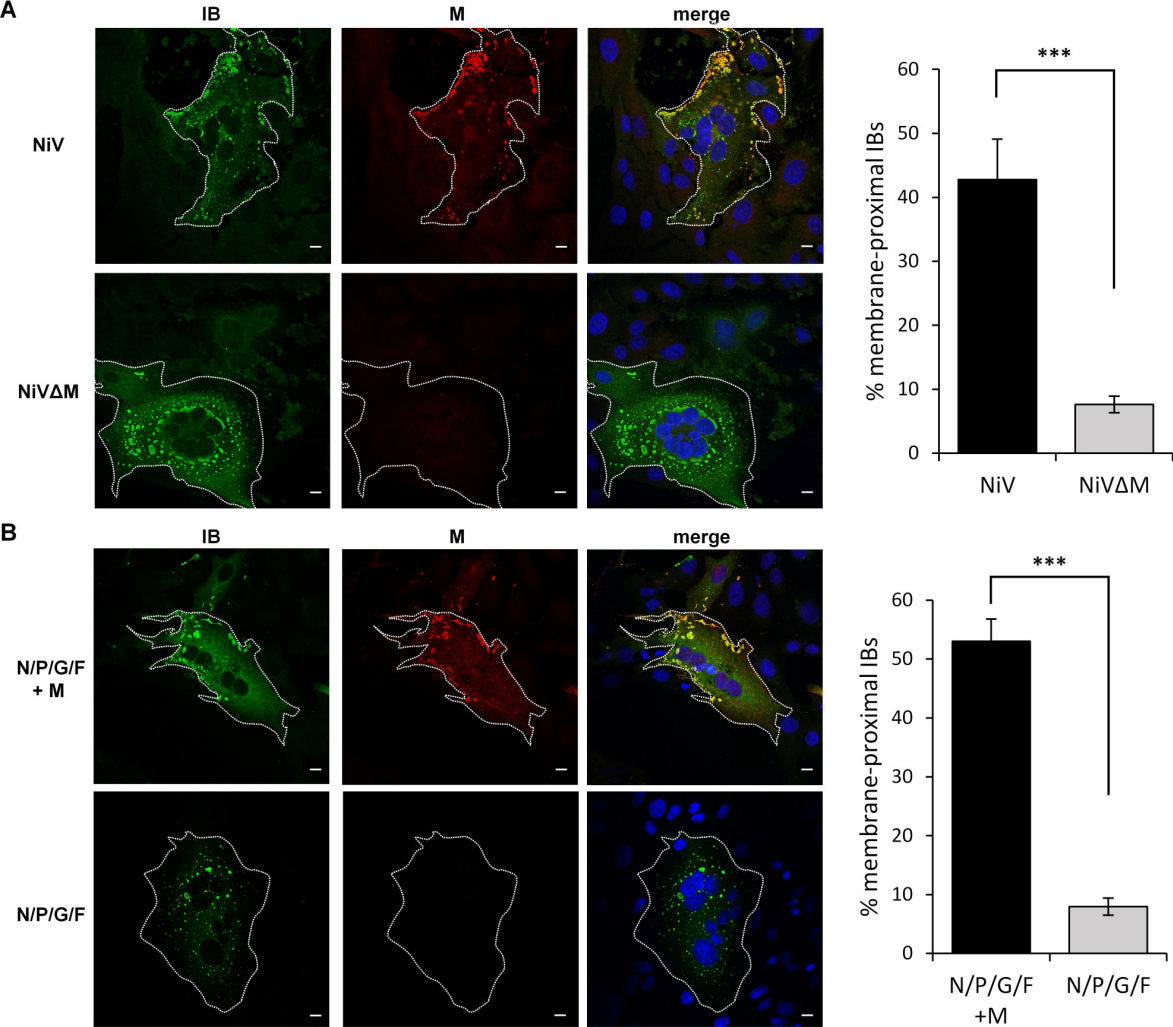
Colocalization of NiV M protein and inclusion bodies in NiV-infected and transfected cells. **(A)** Vero76 cells were infected with wildtype NiV and NiVΔM at a MOI of 0.01 and 0.025, respectively. At 24 h p.i. cells were fixed with 4% PFA for 48 h and permeabilized with Triton X-100. Cells were incubated with a NiV N-specific guinea pig antiserum to visualize IBs (green). NiV M was detected with an M-specific rabbit antiserum (red). Nuclei were counterstained with DAPI (blue). Confocal xy sections of the central regions of syncytia are shown. White dotted lines indicate the lateral borders of syncytia. **(B)** Cells coexpressing the NiV proteins N, P, G and F in the presence (N/P/G/F + M) or absence of NiV M (N/P/G/F) were fixed and permeabilized with Triton X-100 at 24 h after transfection. Immunostaining was performed as described above. Scale bars, 10 μm. In the right panels, quantifications of IB distribution in syncytia is shown. Using the automated ImageJ analyze particle tool, the total numbers of IBs and the number of IBs located at a maximum distance of 10 μm from the lateral edge of the syncytium were counted in individual sections of 6–10 syncytia from three individual experiments. The average percentage of membrane-proximal IBs in syncytia in the absence and presence of M was calculated. Error bars indicate the standard error of the mean. Statistical significance is indicated by asterisks (unpaired t-test; ***, p < 0.001).

The NiV M protein is required for proper NiV particle assembly and budding at the plasma membrane. Since we hypothesized that IB^PM^ represent sites of NiV budding, we next asked if NiV M protein is involved in the formation or localization of these IBs. To test this, we infected cells with recombinant wildtype NiV or M-deleted NiVΔM [16], and analyzed the distribution of IBs 24 h after infection (Fig 2A). A large proportion of the N-containing IBs in syncytia formed by wildtype NiV infection were found in the cell periphery close to the lateral border of the plasma membrane, and the M protein was clearly concentrated in these IB^PM^ (Fig 2A, NiV panels). Some of the M-positive IBs were also found in central regions of the syncytium. These IB^PM^ represent IBs associated with regions of the plasma membrane above the nuclei as shown by analysis of top-to-bottom sections through the syncytium (S3 Fig). In addition to M-positive IB^PM^, N-positive IBs could be detected throughout the cytoplasm, often in perinuclear regions of the multinucleated syncytia. These IB_peri_ did not contain NiV M and were located mostly in middle and bottom sections of the syncytium (Fig 2A, NiV panels; S3 Fig). The pronounced accumulation of the M protein in IB^PM^ but not in IB_peri_ indicated that the two IB subpopulations differ in their subcellular localization as well as in their M protein content, which is a specific "marker" of IB^PM^. Similar M-positive IB^PM^ and M-negative IB_peri_ were also observed in other cell types including NiV-infected bat cells (S4 Fig).

In cells infected with M-deleted NiVΔM, IBs were almost exclusively localized to central and perinuclear regions of syncytia. IBs in membrane-proximal regions were rarely found (Fig 2A, NiVΔM panel). The lack of IB^PM^ demonstrated that the NiV M protein has an essential role in the formation of plasma membrane-associated IBs, and that IBs tend to localize to the region around the cell nucleus in the absence of M. To our knowledge, this is the first demonstration that a matrix protein of a Mononegavirus critically determines the intracellular localization of IBs.

### Expression of the M protein is the primary determinant of IB^PM^ formation

To determine if NiV replication or viral RNA is needed for IB formation or localization, colocalization studies were performed in a cotransfection system. The N and P proteins of other members of the *Mononegavirales* order are the minimal components required for the formation of cytoplasmic IBs [22, 25, 29–31]. Consistent with this, NiV N proteins were shown to interact with cellular RNA forming herringbone-like NC structures, and to recruit wildtype or GFP-tagged NiV P proteins into cytoplasmic IBs [32, 33]. To assess IB formation and localization in the absence of viral infection, we cotransfected varying combinations of plasmids expressing the respective NiV proteins into Vero76 cells and determined their distributions by confocal microscopy. Coexpression of the N, P, and M proteins along with the F and G glycoproteins resulted in a distribution of IB^PM^ and IB_peri_ that reproduced what we observed in cells infected with wildtype NiV (Fig 2B, N/P/G/F + M). IB^PM^ were also associated with the G glycoprotein expressed on the cell surface, supporting the model that IB^PM^ represent viral budding sites (S5A Fig). However, when the M protein was omitted, only IB_peri_ could be detected in transfected cells (Fig 2B, N/P/G/F), and the G protein was more homogeneously expressed on the cell surface (S5B Fig).

**Fig 3:**
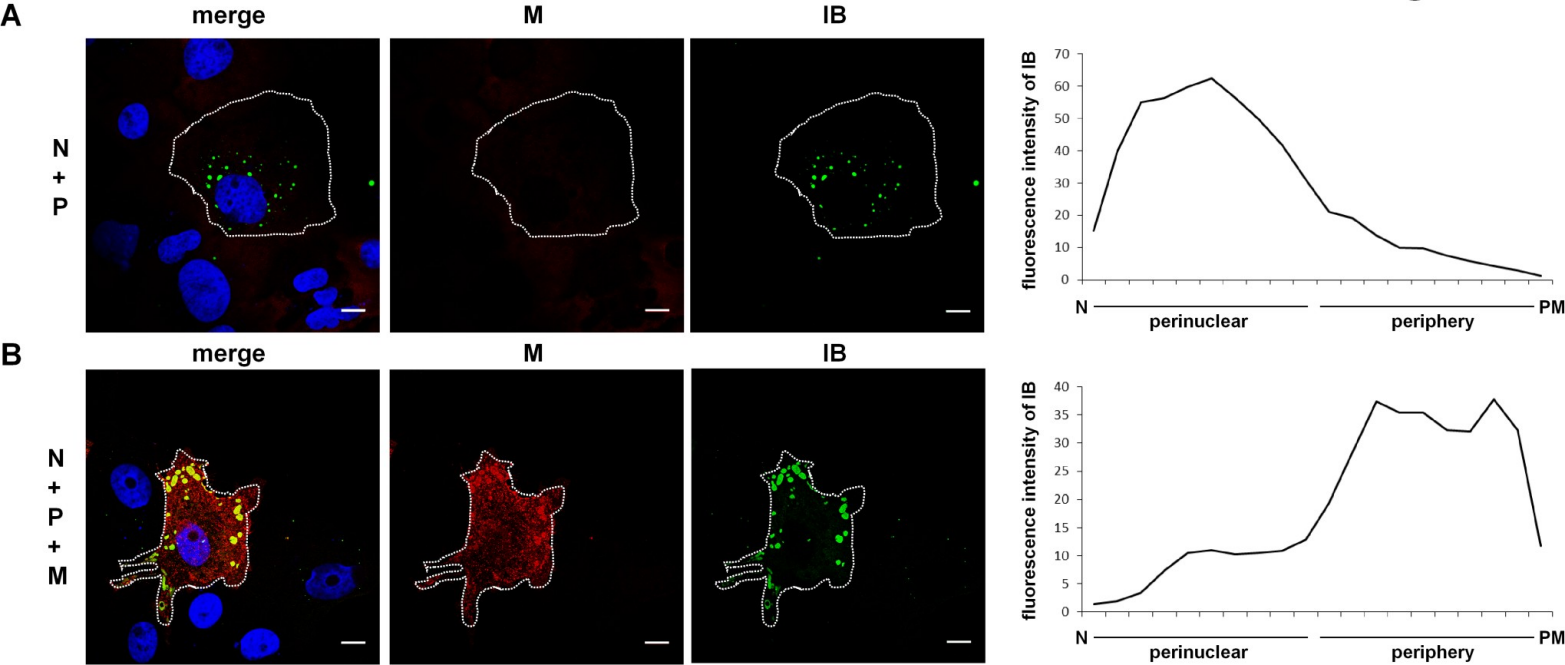
Formation and localization of IBs in the absence and presence of NiV M. **(A)** To induce IB formation plasmid-encoded NiV N and NiV P proteins were coexpressed (N+P). **(B)** N and P proteins were expressed together with the NiV M protein. 24 h after transfection, cells were fixed, permeabilized with Triton X-100 and incubated with an M-specific peptide serum (red, M) and NiV N-specific antibodies (green, IB). Nuclei were counterstained with DAPI (blue). Scale bars, 10 μm. Right panels: To quantify the intracellular localization of IBs (green) in the perinuclear region and the periphery, an ImageJ based quantification tool (IB-LoM) was used. The average IB distribution (quantified in confocal sections of 5–6 cells from three independent experiments) is shown. N, nucleus; PM, plasma membrane.

To determine if M-dependent IB^PM^ formation requires concomitant expression of the surface glycoprotein, or needs ongoing syncytium formation, we omitted the NiV G and F proteins and assessed the distribution of IBs in cells expressing N and P proteins in the absence and presence of the M protein. Expression of the N and P proteins together resulted in the formation of cytosolic IBs in the perinuclear region (Fig 3A). When the M protein was coexpressed with the N and P proteins, inclusions were mostly found in the periphery (IB^PM^) with comparatively few IB_peri_ (Fig 3B). Similar M-positive IB^PM^ and M-negative IB_peri_ were also found in other cells types, such as Huh-7 cells (S6 Fig). Coexpression of the NiV N and P proteins together with M are thus sufficient to induce IB formation at the plasma membrane.

### IB^PM^ form independently at the plasma membrane

**Fig 4:**
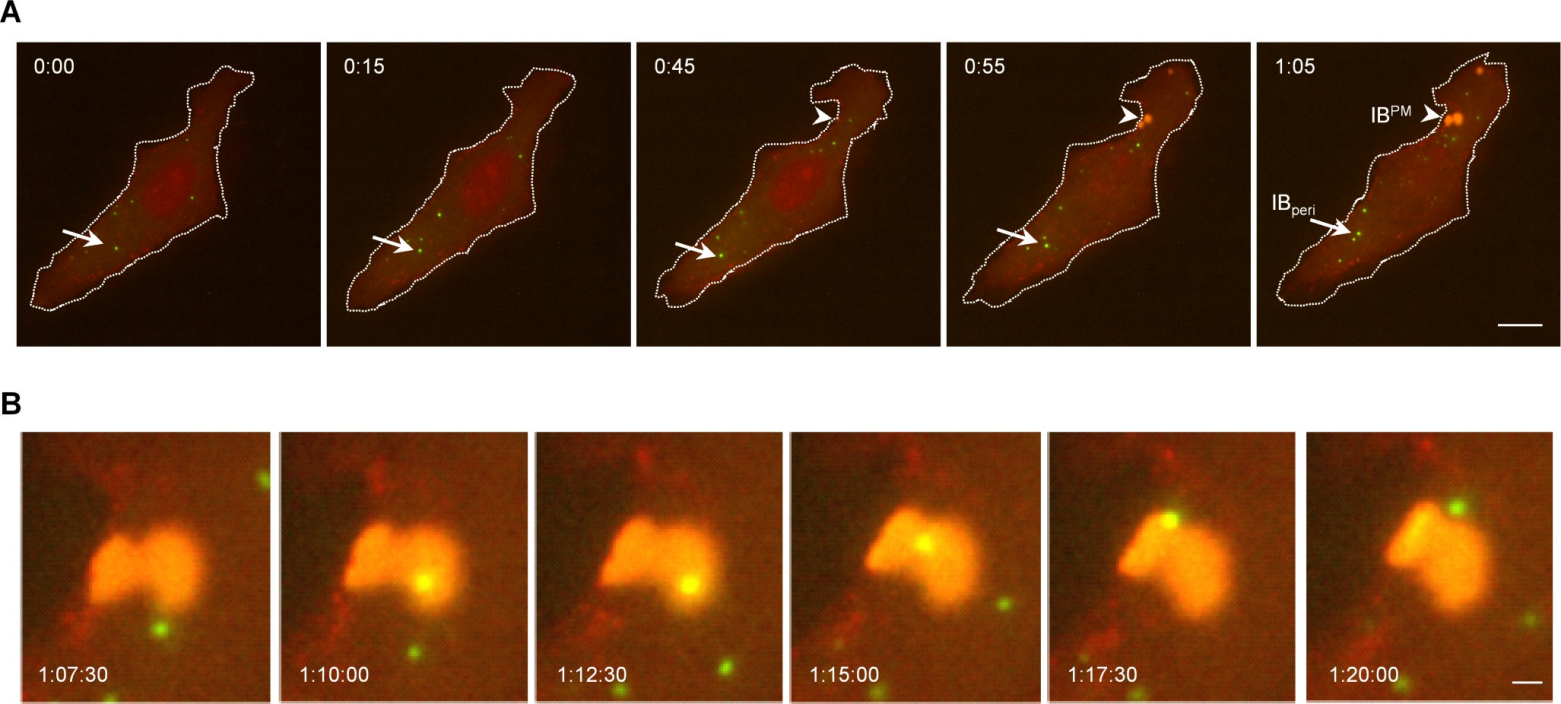
Formation of NiV IB_peri_ and IB^PM^ monitored by live cell imaging. To follow IB formation in the presence of M over time, Vero76 cells were cotransfected with NiV N and NiV P_eGFP_ together with _mCherry_NiV M and untagged NiV M (see Materials and Methods). At 14 h p.t., the live cell time-lapse experiments were started. Images were recorded with a Nikon TE2000 microscope. IBs (green) and M proteins (red) were detected via the P_eGFP_ or the _mCherry_M autofluorescence, respectively. Pictures were taken every 50 sec and processed with Nikon NIS-Elements Microscope Imaging Software. **(A)** Merged images of the whole cell at different time points are shown. The plasma membrane is indicated by the dotted line. Arrows indicate M-negative IBs in the cytoplasm (green). Arrowheads indicate the site where M-positive IBs are formed at the plasma membrane (yellow, IB^PM^). Scale bar, 10 μm. **(B)** Magnification of an IB^PM^ (indicated with an arrowhead in panel a). Scale bar, 1 μm. The full-length video is provided as S1 Movie. For comparison, S2 Movie shows IB formation in the absence of the M protein.

In both infected and transfected cells, M protein-positive IB^PM^ and M protein-negative IB_peri_ could be identified. IB^PM^ may form at the cell surface, or they may originate in another region of the cell and then migrate to the plasma membrane. To answer this question, we investigated the kinetics of IB formation in transfected cells using live cell time-lapse microscopy. Cells were transfected with plasmids encoding the N protein and a P protein tagged with eGFP (P_eGFP_) together with plasmids carrying wildtype and mCherry-labeled NiV M. Small cytoplasmic IBs (shown in green) consisting of only the N and P proteins were formed rapidly and moved readily throughout the cytoplasm (Fig 4A, arrows). These IB_peri_ grew by random fusion with each other in the cytoplasm but did not accumulate at the plasma membrane (S1 and S2 Movies). In contrast, IB^PM^ formed later in close proximity to the plasma membrane (Fig 4A, arrowhead). These IB^PM^ grew in size over time, and contained the M protein from the initial stages of formation (S1 Movie). IB_peri_ can transiently pass IB^PM^, but they were not incorporated into larger IB^PM^ structures (Fig 4B). Perinuclear and plasma membrane-associated IBs are thus independent populations that form with different kinetics. The idea that IB_peri_ form rapidly after N and P protein expression, while IB^PM^ formation starts later because sufficient M protein is required, is also supported by observations in infected cells. We found IB_peri_ in NiV-infected cells at 5 h p.i., while M-positive IB^PM^ were only observed at later times after infection (S7 Fig).

### NiV protein and mRNA synthesis is not confined to inclusion bodies

**Fig 5:**
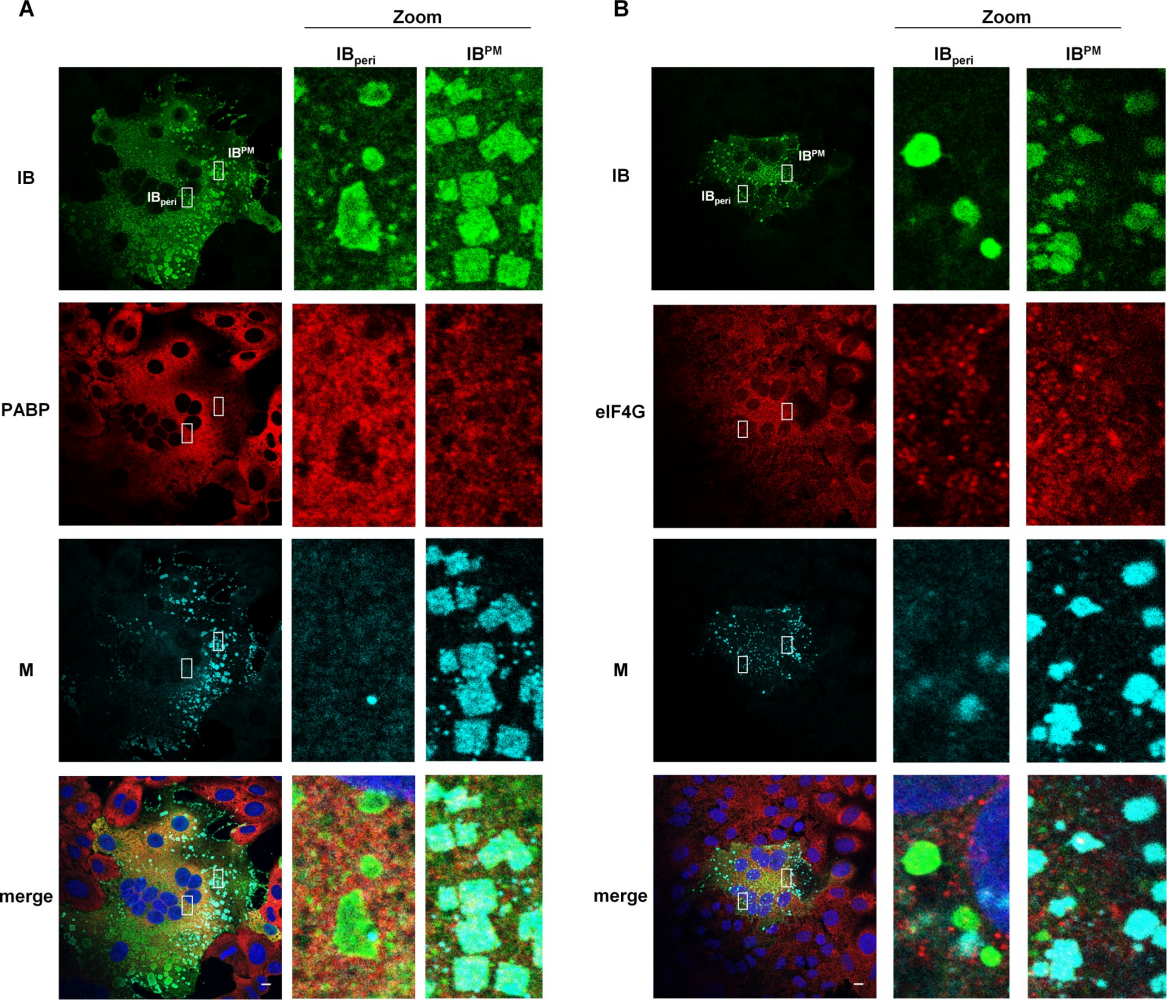
Colocalization of NiV IBs with mRNA binding proteins. Vero76 cells were infected with NiV at a MOI of 0.05. 18.5 h p.i. cells were fixed with 4% PFA for 48 h and permeabilized with methanol/acetone. **(A)** NiV IBs were visualized by using NiV N-specific antibodies (green) and Zenon-labeled anti-M peptide serum (cyan). mRNA (red) was detected with an antibody directed against the PolyA binding protein (PABP). **(B)** NiV IBs (green) and NiV M (cyan) were costained with an anti-eIF4G antibody (red). Nuclei were counterstained with DAPI (blue). In the zoom panels of the confocal images, enlarged views of IB_peri_ and IB^PM^ in the boxed regions are shown. Scale bars, 10 μm.

**Fig 6:**
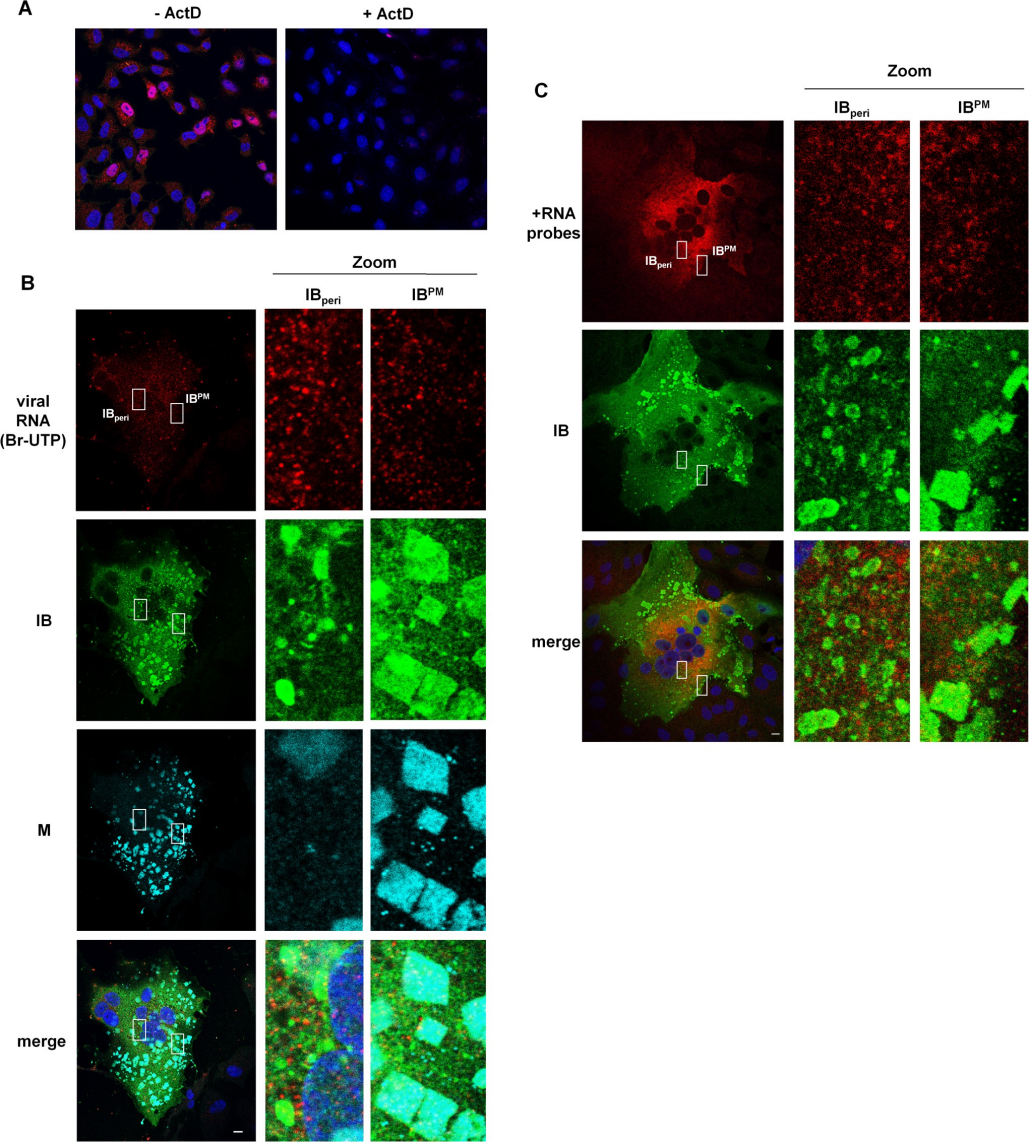
Localization of viral RNA in NiV-infected cells. **(A, B) Colocalization of *de novo* synthesized Br-UTP labeled RNA with IBs.** Vero76 cells were infected with NiV at a MOI of 0.05. 18 h p.i. cells were treated for 1 h with actinomycin D to inhibit cellular transcription or left untreated. Then, cells were transfected with 10 mM Br-UTP. After 20 min, cells were fixed and permeabilized with methanol/acetone. Newly synthesized RNAs were detected using a Br-UTP monoclonal antibody (red). **(A)** Cellular RNA staining in uninfected control cells (Mock) without (-ActD) and with inhibitor (+ActD) are shown. **(B)** In actinomycin D-treated NiV-infected cells, IBs were visualized with an NiV N-specific antiserum (green) and Zenon-labeled anti-M peptide serum (cyan). **(C) Detection of NiV mRNA by FISH.** NiV-infected Vero76 cells were fixed at 18 h p.i. and were probed with Quasar 670-labeled FISH probes targeting the positive-sense NiV RNA from nucleotide 1–6000 (+RNA probes). After hybridization to visualize viral N, P and M mRNA (pseudo-colored in red), the N protein was immunostained to detect IBs (green). Nuclei were counterstained with DAPI (blue). In the zoom panels of the confocal images, enlarged views of IB_peri_ and IB^PM^ are shown. Scale bars, 10 μm.

Many viruses exploit inclusion bodies to concentrate viral proteins and synthesize viral RNA with minimal interference from cellular factors [34]. NiV also accumulates viral proteins and nucleocapsid-like structures in IBs. To address the role of IBs in viral mRNA and protein synthesis, we first assessed whether NiV IBs were associated with proteins involved in mRNA recognition and protein translation. For this we chose poly-A-binding protein 1 (PABP) and eukaryotic initiation factor 4G (eIF4G). In contrast to what has been shown for some other members of the *Mononegavirales* order such as RSV and EBOV [20, 35], both marker proteins were homogenously distributed in NiV-infected cells neither specifically concentrating in IBs, nor in surrounding regions (Fig 5A and 5B). We next determined whether viral mRNA synthesis is concentrated in IBs by labeling NiV-infected cells with Br-UTP in the presence of actinomycin D (ActD) to inhibit cellular transcription. ActD treatment effectively suppressed cellular mRNA synthesis in uninfected control cells (Fig 6A). Viral RNA produced *de novo* during the 20-min labeling period was distributed throughout the cytoplasm but did not accumulate in IB_peri_ or IB^PM^ (Fig 6B). As the amount of genomic RNA compared to the amount of *de novo* synthesized viral mRNA is probably small, the most likely explanation is that the punctuate cytoplasmic RNA staining primarily represents NiV mRNA. Supporting this idea, we found that Br-UTP labeled RNA colocalized with the mRNA binding protein eIF4G (S8 Fig). Consistent with the lack of Br-UTP labeled RNA in either IB_peri_ or IB^PM^, we were also unable to detect viral RNA in IBs if *de novo*-synthesized RNA was labeled with ethynyl uridine (S9 Fig). To confirm the specificity of the cytoplasmic RNA staining detected with Br-UTP labeling, we performed fluorescence *in situ* hybridization (FISH) to visualize NiV mRNAs with a set of 48 Quasar 670 labeled FISH probes [19]. The probes were designed to target the viral positive-sense RNA (+RNA) between nucleotides 1 and 6000 of the NiV genome. These +RNA probes which detect antigenomic RNA and the N, P and M mRNAs, showed a widespread cytoplasmic staining with no concentration in IBs (Fig 6C). Since neither viral mRNA nor PABP and eIF4G proteins associated with translation were concentrated in either IB population, viral RNA and protein synthesis likely occurs in diffuse regions of the cytoplasm before viral proteins are eventually targeted to either IB_peri_ or IB^PM^.

### IB_peri_ likely represent aggresome-like compartments induced by NiV infection

**Fig 7:**
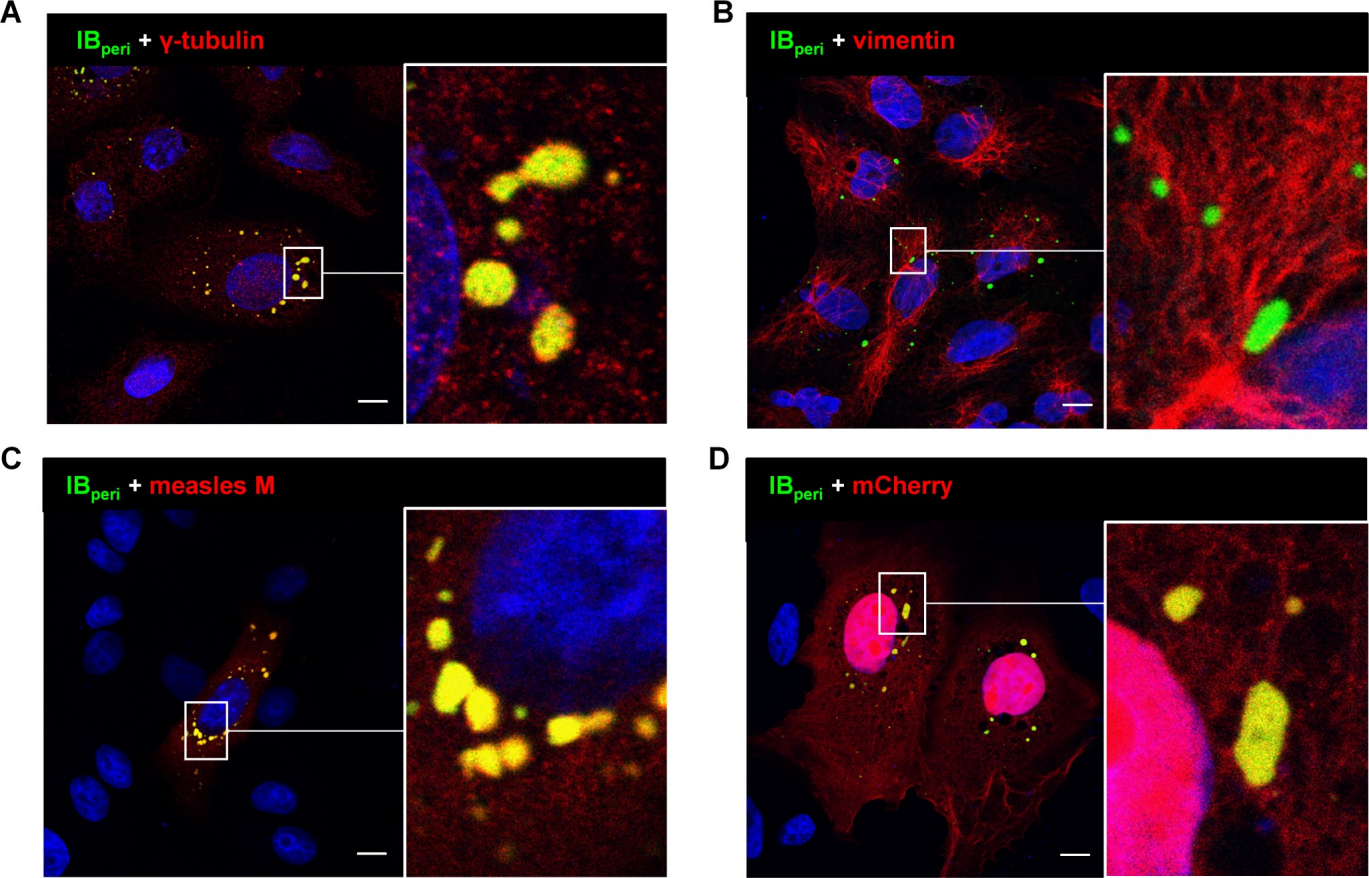
Colocalization of NiV IB_peri_ with cellular aggresome markers and unrelated cytosolic proteins. **(A, B)** Vero76 cells were transfected with NiV N and NiV P_eGFP_ to form IBs (IB_peri_). At 24 h p.t. cells were fixed with 4% PFA and permeabilized with methanol/acetone. IBs were detected by P_eGFP_ autofluorescence (IB_peri_). Cellular aggresome markers (γ-tubulin and vimentin) were detected with specific antibodies (red). **(C, D)** NiV N and NiV P_eGFP_ were coexpressed with non-related cytosolic proteins (red). Measles virus matrix protein (measles M) was detected with a specific monoclonal antibody (C) and reporter mCherry was detected by autofluorescence (D). Nuclei were counterstained with DAPI (blue). Only merged confocal images are shown. IBs within the boxed areas are shown in higher magnification. Scale Bars, 10 μm.

While the function of IB^PM^ likely lies in the assembly of viral NCs and M protein at the plasma membrane to support efficient virus budding, the functional role of IB_peri_ is less evident. Due to their high NC protein content, they resemble cellular aggresomes that can form in response to protein aggregation [36]. We therefore tested whether IB_peri_ colocalize with any other known cellular compartments. We found no evidence in N/P-transfected cells of substantial IB localization to the early endosome (EEA1), late endosome/lysosome (Lamp1), endoplasmic reticulum (ER, calnexin), or Golgi apparatus (GM130) (S10A Fig). In contrast, perinuclear IBs seem to colocalize with γ-tubulin, a central component of cellular and virus-induced aggresomes [36, 37] (Fig 7A; S11A Fig). Although we did not observe any changes in the vimentin arrangement around IBs (Fig 7B), IB_peri_ showed the characteristic ability of cellular aggresomes to recruit overexpressed cytoplasmic proteins. This is exemplified by the staining of coexpressed measles virus (MeV) matrix protein (Fig 7C) and reporter mCherry (Fig 7D, S11B Fig), both of which are unrelated to NiV but clearly colocalized with NiV IB_peri_. To determine if IB_peri_ also share components with cellular stress granules, we performed costaining experiments with the stress granule marker G3BP1, but found no evidence of its colocalization with IB_peri_ (S10B Fig). IB^PM^ did not contain y-tubulin nor did they recruit the reporter mCherry (S12 Fig), which confirms the substantial differences between IB_peri_ and IB^PM^.

**Fig 8:**
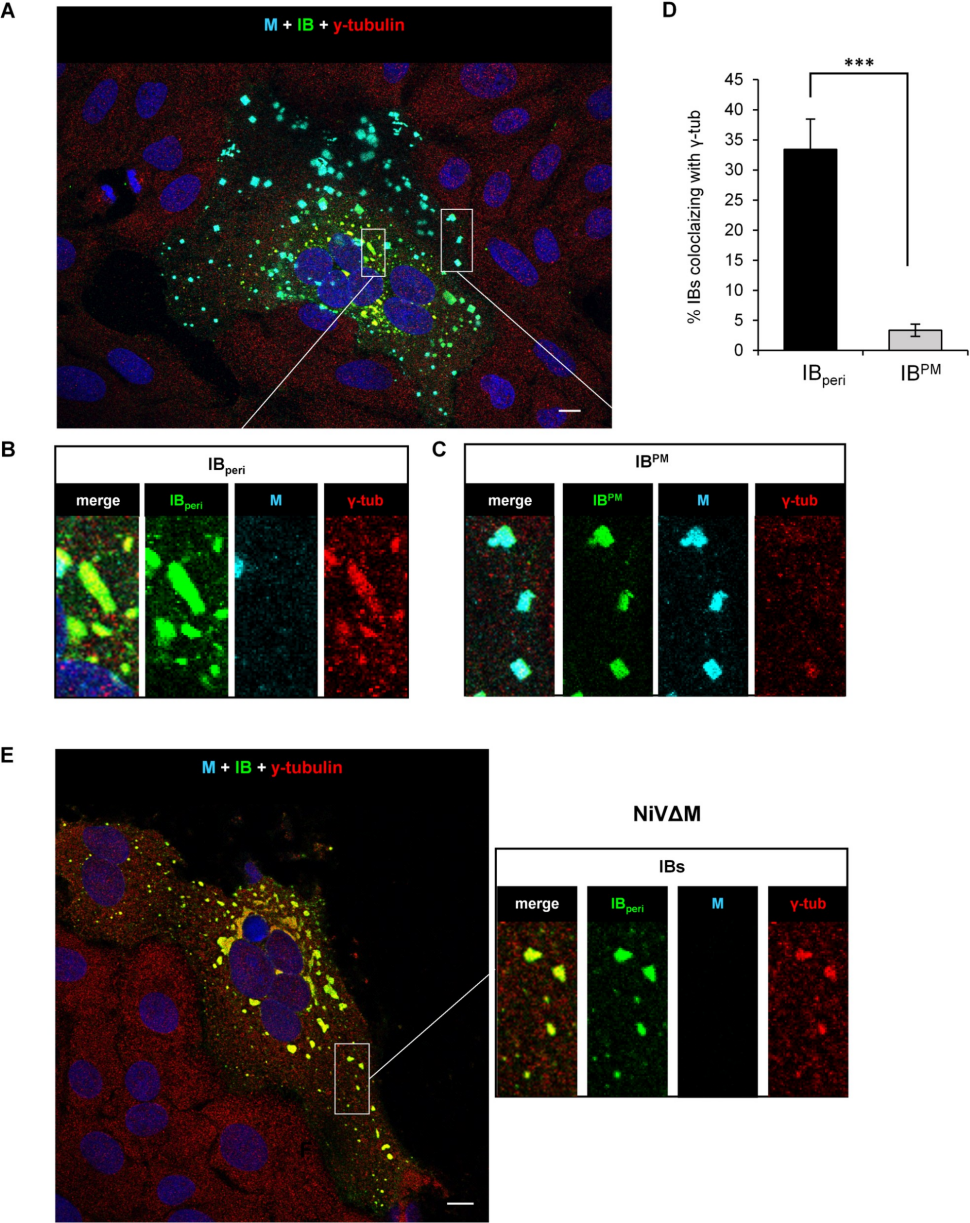
Colocalization of NiV inclusion bodies with y-tubulin in virus-infected cells. Vero76 cells were infected with NiV or NiVΔM at a MOI of 0.05 or 0.01, respectively. At 18.5 h p.i. cells were fixed with 4% PFA for 48 h and permeabilized then with methanol/acetone. Cells were stained with an anti-N serum to visualize IBs (green) and with a Zenon-labeled anti-M peptide serum (cyan) to identify M-positive IB^PM^. For γ-tubulin detection, cells were incubated with anti-γ-tubulin mouse antibodies (red). **(A)** A confocal section through a NiV-induced syncytium is shown. Magnifications and individual staining of the boxed areas are presented in the bottom panel. **(B)** M-negative and y-tubulin positive IB_peri_. **(C)** M-positive and y-tubulin negative IB^PM^. **(D)** Colocalization of M-positive IB^PM^ and M-negative IB_peri_ with y-tubulin in NiV-induced syncytia (n = 8) was quantified using the ImageJ-based macro IB-Coloc. Error bars indicate the standard error of the mean. Statistical significance is indicated by asterisks (unpaired t-test; ***, p < 0.001). **(E)** A confocal section through a NiVΔM-induced syncytium is shown. The right panel shows a magnification and individual staining of the IBs in the boxed area.

Finally, to strengthen the conclusion that IB_peri_, but not IB^PM^, represent a virus-induced aggresome-like compartment, we analyzed the colocalization of both IB populations with y-tubulin in NiV-infected cells. Numerous IB^PM^ and several IB_peri_ were found in NiV-infected cells 18.5 h after infection (Fig 8A). As shown in the image magnifications, y-tubulin was clearly concentrated in IB_peri_ devoid of M protein (Fig 8B), whereas y-tubulin was virtually absent in M protein-containing IB^PM^ (Fig 8C and 8D). NiV infection thus induces two unrelated IB populations, one of which contains the M protein and is associated with the plasma membrane and virus budding (IB^PM^), while the other forms M-negative but y-tubulin positive aggresome-like structures often located around the nucleus (IB_peri_). In NiVΔM-infected cells, the large IBs substantially colocalized with y-tubulin (Fig 8E) confirming that IB_peri_ are formed in the absence of the M protein.

## Discussion

Cytoplasmic IB formation is a hallmark of infection by members of the *Mononegavirales* order. Studies in recent years have revealed that IB composition, spatio-temporal requirements for IB formation, and their exact function can differ substantially, even between closely related viruses. We show here that NiV infection induces two types of IBs: one population in the perinuclear region (IB_peri_) that are formed rapidly upon expression of the N and P proteins; and another population whose formation in the cell periphery in proximity to the plasma membrane (IB^PM^) is dependent on the additional expression of the M protein. IB_peri_ were found to be positive for y-tubulin and they recruited overexpressed cytoplasmic proteins, and therefore share some characteristics with aggresomes. In contrast, IB^PM^ are associated with the plasma membrane and appear to form a platform where virus assembly and budding may be facilitated.

### NiV transcription occurs in the cytoplasm rather than in cytoplasmic inclusion bodies

Cytoplasmic IBs contain NC-like structures and large amounts of viral proteins. However, most of the viral genomic and antigenomic RNA, as well as viral mRNA were found to be loosely distributed in a network of membrane-like reticular structures that were often in close proximity to the RER [28]. These reticular structures were proposed to represent sites of viral replication and transcription; an idea that corresponds with our detection of NCs outside of IBs (S2 Fig) and the findings that no detectable *de novo* synthesized viral mRNA or cellular proteins involved in translation initiation accumulated in IBs. We found that even with short labelling times, newly-synthesized RNA was not associated with IBs, which conflicts with the idea that the detection of viral mRNA in the cytoplasm was due to rapid export of mRNA from IBs and subsequent spread throughout the cytoplasm. This seems unlikely because the polymerases of *Mononegavirales* order members are assumed to synthesize mRNA with a transcriptional elongation rate of approximately 3 nucleotides per second [38, 39]. At that rate, it would take longer than 20 min for viral mRNAs to be produced *de novo*. If transcription occurs in IB_peri_, a partial accumulation of Br-UTP labeled NiV RNA would be expected in inclusions, but this was not the case. Though NiV genome replication in the cytoplasm has not yet been directly demonstrated, the lack of +RNA detection in the form of mRNA and viral antigenomic RNA in IBs indirectly suggests that not only transcription but also genomic RNA synthesis occurs outside of IBs.

### Role of IB_peri_ in NiV infection

While IB_peri_ are not major sites of NiV RNA synthesis, they are likely of functional importance. As an aggresome-like compartment they can passively accumulate large quantities of overproduced viral proteins, thereby preventing toxicity to the cell. They may also function as storage compartments for NCs that are not directly transported to the plasma membrane. In this scenario, NCs and viral RNA would be sequestered in the cytoplasm and shielded from cytoplasmic nucleic acid sensors so that they do not trigger innate cellular immune responses [40, 41]. It is not clear if these compartments are terminal destinations for NCs, or if they would function more as temporary storage for NCs before they are recruited to the plasma membrane for incorporation into IB^PM^. Based on studies with other members of the *Mononegavirales* order, it has also been proposed that cytoplasmic inclusions are not insoluble aggregates of viral proteins but rather have properties similar to liquid compartments or organelles [23, 42, 43]. The NiV IB_peri_ that we observed were highly mobile spherical structures that readily fused with each other to form larger spheres, which would fit with this model. In addition, the IBs in HMPV-, RABV-, and VSV-infected cells do not contain their respective M proteins [19, 21, 23], which is similar to what we observed for IB_peri_ in NiV infection. However, it remains to be determined if the mobile and fusogenic character of IB_peri_ we observed in cells expressing N and P proteins (S1 and S2 Movies) actually reflect the properties of IB_peri_ in NiV-infected cells in which replication is ongoing and additional viral RNA and proteins are present. Although the potential liquid structure of IB_peri_ requires further studies in infected cells, their properties clearly differ from the second IB subpopulation. IB^PM^ were formed later in either transfected or infected cells (see Figs 4 and S7), contained large amounts of the M protein, are pleomorphic, sometimes even squarish, and did not move and fuse with each other in the cytoplasm.

### A model for inclusion formation and NiV assembly

**Fig 9:**
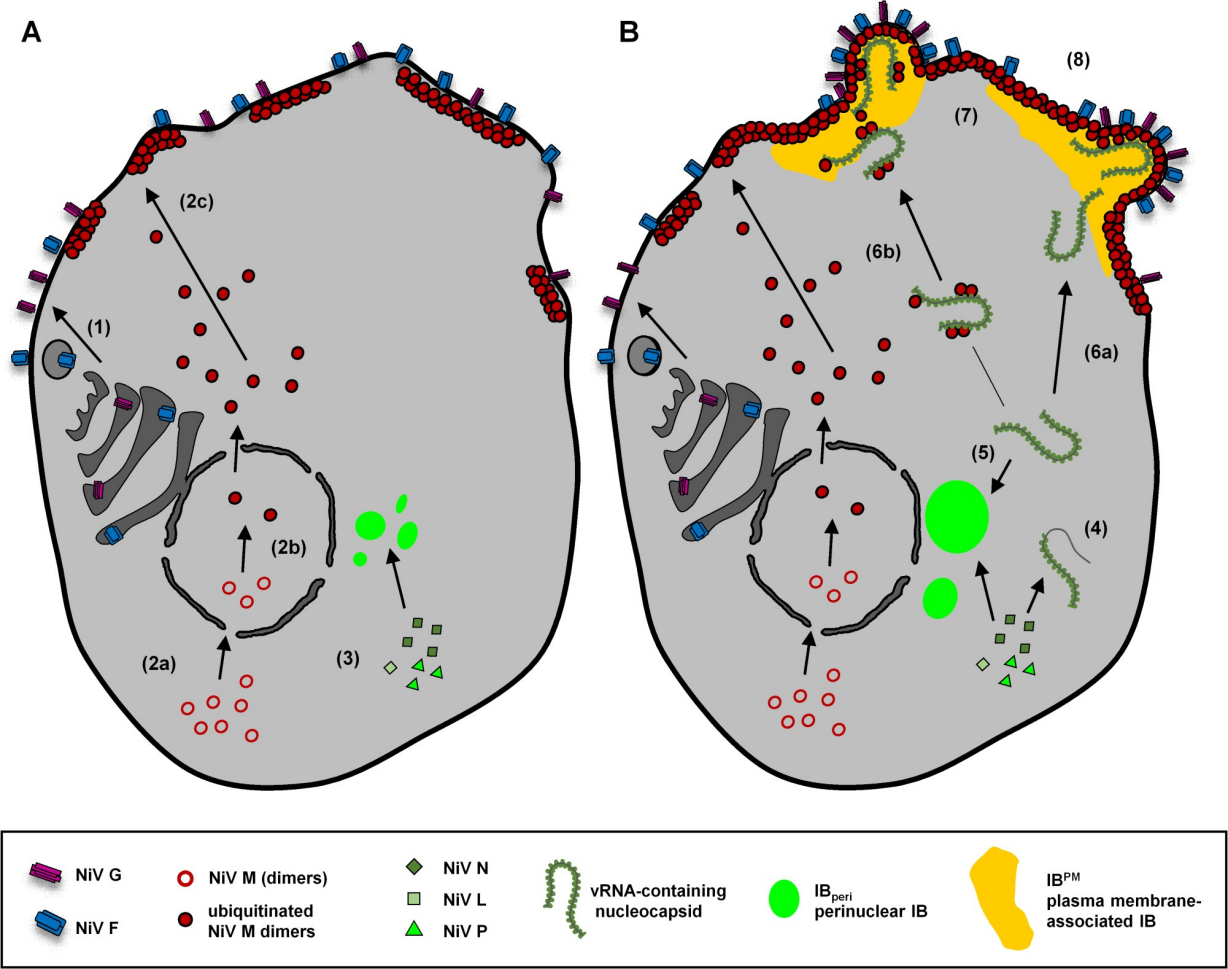
Model for IB formation and assembly during NiV infection. **(A)** NiV protein synthesis and trafficking in early infection stages. NiV surface glycoprotein F and G are synthesized in the ER and transported to the plasma membrane via the secretory pathway **(1)**. NiV M is synthesized in the cytoplasm and rapidly imported into the nucleus **(2a)**. Only after monoubiquitination at K258, NiV M can exit the nucleus **(2b)**. After nuclear transit, NiV M traffics to the plasma membrane to form a dense matrix underneath the plasma membrane **(2c)**. NiV N, P, and L proteins are synthesized at ribosomes throughout the cytoplasm and concentrate rapidly in perinuclear IBs **(3)**. **(B)** IB formation and assembly in late infection stages. NiV replication and transcription is assumed to occur in cytoplasmic reticular structures rather than in IBs. Newly synthesized viral genomes are encapsidated by the viral nucleocapsid proteins to form NCs **(4).** NCs are recruited to IB_peri_ until sufficient M protein is expressed **(5)**. NC are transported to the cell periphery either independently **(6a)** or via cotransport with M proteins **(6b)**. NCs and M proteins accumulate at the plasma membrane and form IB^PM^ **(7)**. The NiV glycoproteins are clustered at IB^PM^ sites and virus budding is initiated **(8)**. Please refer to the text for a more detailed discussion of the model.

Based on the results of this study and earlier studies on NiV replication, we propose the following model for IB formation and assembly of NiV particles at the plasma membrane. Fig 9 schematically illustrates the steps in early (Fig 9A, steps 1–3) and late NiV infection (Fig 9B, steps 4-8).

Shortly after successful virus entry, viral mRNAs are synthesized and NiV-infected cells begin to produce viral nucleocapsid- and envelope-associated proteins. The NiV glycoproteins G and F are synthesized in the rough endoplasmic reticulum and are transported via the Golgi apparatus to the plasma membrane (Fig 9A, step 1). After the NiV F protein is internalized by endocytosis and is proteolytically activated by endosomal cathepsins, fusogenic NiV glycoprotein complexes are constitutively expressed on the cell surface and can initiate cell-cell fusion [4, 44, 45]. The M proteins are translated at free ribosomes in the cytoplasm (Fig 9A, step 2a), are then imported into the nucleus and are ubiquitinated at lysine 258 [11] (Fig 9A, step 2b). After export from the nucleus, M proteins migrate to the plasma membrane, although the precise pathway, which might involve AP-3 [8], remains to be elucidated. Formation of a grid-like array is characteristic for all paramyxovirus M proteins that typically exist as dimers and polymerize at the plasma membrane [46, 47]. NiV M proteins thus form a dense protein matrix underneath the plasma membrane (Fig 9A, step 2c). The NiV nucleocapsid proteins N, P, and L are also synthesized at cytoplasmic ribosomes. In contrast to M, these viral proteins rapidly concentrate in perinuclear inclusions (Fig 9A, step 3).

Later in NiV infection, transcription and protein synthesis is accompanied by genome replication, NC formation, and other assembly processes, which are depicted in Fig 9B. As with viral mRNA, viral genomic RNA is assumed to be synthesized at reticular structures in the cytoplasm [28]. Newly transcribed NiV genomes are rapidly encapsidated by nucleocapsid proteins (Fig 9B, step 4) and are recruited to IB_peri_ (Fig 9B, step 5). In the absence of M, the sizes of IB_peri_ can be very large, such as those we have observed in NiVΔM-infected cells (see Fig 2A, NiVΔM panel, Fig 8E). If sufficient M protein is present, vRNA-containing NCs are recruited to the plasma membrane. NCs formed in the cytoplasm may directly traffic to viral assembly sites at the cell surface. However, it cannot be excluded that NiV NCs are first recruited to IB_peri_, and then later egress and migrate to participate in IB^PM^ formation. Such an exit of viral nucleocapsids from cytoplasmic inclusions has been demonstrated for RABV and MARV [43, 48]. Eventually, NiV NCs accumulate in IB^PM^ that, at later stages of NiV infection, exceed IB_peri_ in numbers and sizes (see Fig 2A, NiV panel). The recruitment of NCs to the plasma membrane depends on the M protein, although the trafficking route that NCs follow in their journey to the cell surface, has yet to be determined. Similar to what has recently been proposed for measles virus [49], NiV NCs could be transported through the cytoplasm independently from the M protein, and only come into contact and interact with the M protein as part of the pre-formed grid-like matrix structure at the plasma membrane (Fig 9B, step 6a). Alternatively, NCs might already bind to the M protein in the cytoplasm and reach the plasma membrane via a co-transportation mechanism (Fig 9B, step 6b). Independently from the trafficking route, NCs associated with M proteins accumulate and form large inclusions underneath the plasma membrane (Fig 9B, step 7) at which the viral glycoproteins are clustered and budding of NiV particles is initiated (Fig 9B, step 8).

We have demonstrated here that IBs formed in cells infected with NiV differ from those of other *Mononegavirales*. We show that distinct populations of IBs associated with the perinuclear region and the plasma membrane are produced. The strict dependence of plasma membrane-associated IB^PM^ on the M protein strongly suggests a functional role in virus assembly and budding. Our data highlight the importance of investigating the determinants of IB formation for diverse members of the *Mononegavirales* order as well as their spatio-temporal distributions to expand the applicability of available models describing different aspects of viral life cycles. It also provides a starting point for the development of novel therapeutic approaches with the aim of disrupting processes driving the formation and development of inclusion bodies in infections with highly pathogenic NiV.

## Materials and methods

### Reagents and antibodies

All cell culture media and supplements were supplied by Gibco, (Eggenstein, Germany). Triton X-100, actinomycin D (ActD), 5-Bromouridine 5′-triphosphate sodium salt (Br-UTP), and paraformaldehyde (PFA) were all purchased from Merck. Stock solutions of ActD (1 mg/ml) and Br-UTP (100 mM) were prepared in water. 4% PFA was prepared in DMEM. Methanol and acetone supplied by Riedel-de Haen. The Click-iT RNA Alexa Fluor 488 Imaging Kit, the Zenon Alexa Fluor 647 Rabbit IgG Labeling Kit and DAPI (4´,6-diamidino-2-phenylindole) were supplied by ThermoFisher. Mowiol 4–88 was supplied by Calbiochem. 1,4-diazabicyclo(2,2,2)octane (DABCO) and 6-hydroxy- 2,5,7,8-tetramethylchromane-2-carboxylic acid (Trolox) were purchased from Sigma-Aldrich. The guinea pig anti-serum against the NiV N protein (GP3; 1:500) was kindly provided by Heinz Feldmann (Rocky Mountain Laboratory, NIH). The rabbit peptide serum against the NiV M protein (IG1321; 1:250) was generated by ImmunoGlobe. The mouse antibodies against eIF4G (sc-133155; 1:50) and vimentin (SC-6260; 1:50) were supplied by Santa Cruz; mouse antibodies against early endosome antigen 1 (EEA1; 610456; 1:50), Lamp1 (611042; 1:50), GM130 (610823/22; 1:30), G3BP1 (611127; 1:200) were supplied by BD Transduction Laboratories. The mouse anti-bromodeoxyuridine (clone BMC9318; 1:20) and rabbit anti-γ-tubulin (T3559; 1:1000) antibodies were supplied by Merck. The mouse anti-HA (16B12; 1:200) antibody was supplied by Biolegends, and the mouse anti-Calnexin (ab31290; 1:100) and rabbit anti-PABP1 (ab21060; 1:200) antibodies were supplied by Abcam. The mouse anti-measles virus matrix protein (MAB8910; 1:100) was supplied by Chemicon. Secondary antibodies raised against mouse, goat, guinea pig and rabbit IgG and conjugated to Alexa Fluor 488 or 568 were supplied by Invitrogen.

### Cells and virus infections

Vero76 cells (CRL-1587, ATCC), human hepatoma cells (Huh-7, RRID:CVCL_0336) and fruit bat kidney cells from Eidolon helvum (EidNi/41.3; RRID: CVCL_RX14) [50] were cultivated in Dulbecco’s modified Eagle’s medium (DMEM) with 2–10% FCS, 100 U penicillin ml^−1^, and 0.1 mg streptomycin ml^−1^ and 4 mM L-glutamine.

All infection experiments with live viruses were performed under biosafety level 4 (BSL-4) conditions at the Institute of Virology, Philipps University Marburg. The NiV isolates used in this study, recombinant NiV_Malaysia_ (NiV) and M-deleted NiV (NiVΔM), have been described previously [16]. For infection of confluent Vero76 cells, cell cultures were incubated with NiV or NiVΔM at an MOI of 0.01 to 2 for 1–2 h at 37°C. After virus adsorption, cells were washed five times with PBS supplemented with MgCl_2_ and CaCl_2_ (PBS^++^) and then incubated in DMEM with 2% FCS at 37°C.

### Plasmids

pCG and pCAGGS-based expression plasmids encoding NiV F, NiV G_HA_ and NiV M have been previously described [51, 52]. To generate a N-terminal mCherry-fusion protein, the mCherry sequence with a (glycine-serine)_6_-linker sequence was inserted in-frame into pCG NiV M by overlapping PCR (_mCherry_NiV M). For the generation of NiV N and P expression plasmids, we used pTM1 plasmids containing the untagged versions of the NiV N and NiV P genes [16]. NiV N and NiV P sequences were amplified by PCR and inserted between the NotI and PacI sites of a pCG vector with the multiple cloning site of pMCS5 (pCG-MCS). To construct a fluorescently labeled P protein, a N-terminal GFP fusion protein was generated. For this, the eGFP coding sequence was inserted in-frame with the NiV P gene and subcloned into pCG-MCS using the NotI and PacI sites (NiV P_eGFP_). pCAGGS-mCherry was kindly provided by Cornelius Rohde who cloned the mCherry gene into the pCAGGS vector using the Xho1 and Nhe1 restriction sites.

### Electron microscopy

For transmission electron microscopic analysis cells were grown in 6-wells plates and were infected with recombinant wildtype NiV at an MOI of 2. At 24 h post-infection (p.i.) cells were fixed with 4% PFA and 0.1% glutaraldehyde in 0.1 M PHEM buffer [60 mM piperazine-N,N = -bis(2-ethanesulfonic acid) (PIPES), 25 mM HEPES, 2 mM MgCl_2_, 10 mM EGTA (pH 6.9)] for 30 min at room temperature. The cells were then scraped and were pelleted by centrifugation for 10 min and 13,000 rpm at 4°C. The supernatant was discarded and the cells were then overlaid with 4% paraformaldehyde. Following an established protocol [53], cell pellets were washed with 0.1 M cacodylate buffer and postfixed with 1% OsO_4_ in 0.1 M cacodylate buffer containing 0.05 M potassium ferricyanide for 1 h on ice. After washing with cacodylate buffer, cells were incubated with 2% aqueous uranyl acetate solution for 2 h at room temperature in the dark, dehydrated in graded ethanol solutions, embedded in a mixture of Epon and Aralite, and polymerized at 60°C for 24 h. Ultrathin sections (60–90 nm) of the cells were cut with a Leica EM UC6 ultramicrotome. The sections were contrasted with uranyl acetate and lead citrate. The samples were analyzed with a JEM 1400 transmission electron microscope at 120 kV. Images were acquired using a TVIPS TemCam F416 camera.

### Confocal microscopy and double immunofluorescence staining analysis

For immunostaining of NiV-infected cells, Vero76 or EidNi/43.1 cells were grown on glass coverslips in 24-wells plates and were infected with NiV or NiVΔM. At 18.5 to 24 h p.i., infected cells were inactivated for 48 h with 4% PFA, removed from the BSL-4 facility and further processed under BSL-2 conditions. To block the PFA, cells were first incubated with DMEM containing 10% FCS for 1 h, followed by incubation with 0.1 M glycine in PBS^++^ for 15 min. After washing, cells were permeabilized with 0.1% Triton X-100 in PBS^++^ for 15 min or methanol/acetone (1:1) for 5 min. For coexpression studies, Vero76 or Huh-7 cells were transfected with Lipofectamine 2000 and the respective plasmids encoding the NiV M, N, P_eGFP_, P, F, and G_HA_ proteins. Cells were fixed after 24 h with 4% PFA and subsequently permeabilized with 0.1% Triton X-100 or methanol/acetone (1:1). For immunostaining, cells were labeled with the respective primary antibodies for 60 min followed by appropriate Alexa Fluor-conjugated secondary antibodies for 60 min. Antibodies were diluted in PBS^++^ containing 0.35% bovine serum albumin. The cell nuclei were counterstained with DAPI. The samples were mounted in Mowiol 4–88 containing 10% DABCO and examined using a Leica confocal laser scanning microscope SP5.

### Quantification of IB distribution in confocal images of multinucleated syncytia

To determine the percentage of IBs localized in central and peripheral regions of a syncytium, we used standard tools and plugins of the open source software ImageJ (v.1.51). Using standard selection tools, we defined the membrane-proximal region (ROI_memprox_) as a 10 μm-thick boundary beneath the cell borders. Next, N-positive IBs were segmented employing the following tools and settings: Auto Local Threshold (Phansalkar, radius 50), Median filter (radius 2), Watershed, Analyze Particles (size ≥ 1 μm^2^). Comparison with the original images verified the correct recognition of IB structures. Subsequently, we counted all N-positive IBs within ROI_memprox_ and in the whole syncytium, respectively. The ratio of total IBs and IBs within the 10 μm boundary determined in at least 6 different syncytia finally gave the relative proportion of IBs in this plasma membrane proximal region in the absence and presence of the M protein.

### Quantification of confocal image data of single cells with IB-LoM

To quantify localization of NiV IBs in perinuclear and peripheral cell regions in cells with a single nucleus, an ImageJ-based macro was developed. The IB-LoM macro automatically quantifies the fluorescence intensity of target objects (e.g. inclusion bodies) and measures their relative distance to the nucleus and the plasma membrane in a manner similar to the border-to-border method [54]. For the data collection, we used stacks containing images of the DAPI, NiV M (red), and NiV P_eGFP_ (green) fluorescence, and defined two regions of interest (ROIs) for each individual cell. The selection tools in ImageJ software were used to define the border of the nucleus from the DAPI image, and the edges of the cell from an image of the cytoplasmic NiV M staining. Both ROIs were determined with the automatic thresholding function combined with a median filter. In the event that automatic thresholding failed (e.g., because of contact with neighbouring cells), ROIs were manually adjusted using the standard ImageJ software selection tools. After setting the ROIs, at least 600 radial lines starting at the nucleus border and ending at the plasma membrane were automatically drawn. Lines which crossed more than two borders were excluded from the analysis. The data collected for the pixels along each line in the P_eGFP_ image (representing the IBs) were (i) the line number, (ii) the mean fluorescence intensity, and (iii) the relative (fractional) distance between the nucleus and the edge of the cell. For the fractional analysis, each line was divided into 20 fractions with the same length, each representing 5% of the total distance from the nucleus to the plasma membrane. The first 10 fractions (0–50%) were defined as perinuclear regions; and the last 10 fractions (55–100%) were defined as peripheral regions. For every 5% fraction, the mean fluorescence intensity was calculated and plotted against the corresponding relative distance to the nucleus. To focus the analysis to IBs, a minimum threshold value of 15 was defined. Lines crossing an IB contain intensities surpassing this threshold in at least one fraction. These were considered as “object lines” and included in the analysis. Requests for the IB-LoM macro code should be addressed to SH (halwes@staff.uni-marburg.de).

### Live cell imaging

Previous reports of Marburg and Ebola viruses have described that viral proteins fused to larger fluorescent tags were only fully functional if the respective wildtype proteins are also expressed [48, 55]. Similarly, NiV M tagged with mCherry was functional regarding membrane association, and virus-like particle induction, but only interacted and colocalized with N protein and IBs in the presence of the untagged wildtype NiV M protein. Therefore _mCherry_M and wildtype M were always coexpressed at a ratio of 1:5. For live cell imaging, Vero76 cells were grown in 35-mm μ-Ibidi dishes and transfected with the respective plasmids encoding the _mCherry_M, wildtype M, N, and P_eGFP_ proteins using FuGene HD. At 14 h p.t. medium was replaced by CO_2_-independent Leibovitz’s medium without phenol red with 10 % FCS, 100 U penicillin ml^−1^, 0.1 mg streptomycin ml^−1^, 4 mM L-glutamine and 1 mM Trolox. Live cell time-lapse experiment images were recorded with a Nikon TE2000 microscope using a 63x oil objective. Pictures were taken every 50 sec and processed with Nikon NIS-Elements Microscope Imaging Software.

### Triple immunostaining using Zenon-labeled anti NiV M antibodies

For triple staining, such as the costaining of γ-tubulin, NiV-G_HA_, PABP1, eIF4G, Br-UTP or EU and NiV M, anti-M antibodies were directly labeled using a Zenon Labeling Kit. NiV-infected cells were fixed with 4% PFA and permeabilized with methanol/acetone (1:1) for 5 min or 0.1% Triton X-100 in PBS^++^ for 15 min. Cells were then labeled with rabbit antibodies directed against y-tubulin (or mouse antibodies detecting G_HA_, PABP, eIF4G or Br-UTP), and secondary antibodies conjugated to Alexa Fluor 568. Then cells were incubated with a 5% preimmune serum from rabbit for 1 h. NiV M specific rabbit antibodies (IG1321) were labeled with Zenon647 according to the manufacturer’s instructions, and added to the cells in a dilution of 1:20 for 1 h. After washing, IBs were detected with anti-NiV N guinea pig antibodies (GP3) and Alexa Fluor conjugated secondary antibodies. The samples were mounted and examined by confocal microscopy.

### Quantification of colocalization of y-tubulin and IB^PM^ and IB_peri_ with IB-Coloc

For quantifying the colocalization between y-tubulin and the different IB populations in virus-induced syncytia we employed an object-based quantification method. Briefly, NiV-infected Vero76 cells were immunostained for NiV N protein (green), NiV M protein (cyan) and y-tubulin (red) as described above and IB recognition was conducted as described above for the quantification of IB distribution. Next, M-negative IBs (IB_peri_) were separated from IB^PM^ positive for both, M and N with an overlap of at least 50%. Then, y-tubulin containing IBs were identified by applying the same object parameters (except for reducing the threshold radius to 15). IB^PM^ and IB_peri_ showing an overlap of at least 50% with y-tubulin objects were considered y-tubulin positive IBs. After that, the number of y-tubulin positive IBs within each IB subpopulation was determined to give the percentage of y-tubulin positive IB^PM^ and IB_peri_, respectively. Requests for the IB-Coloc macro code should be addressed to SH (halwes@staff.uni-marburg.de).

### Surface staining of the NiV G glycoprotein

Vero76 cells were grown on glass coverslips in 24-wells plates and were transfected with the respective plasmids encoding the N, P_eGFP_, M, F, and G_HA_ proteins. Since extensive syncytia formation can interfere with surface immunostaining in unfixed samples, cell-cell fusion was blocked by adding 20 mM ammonium chloride (NH_4_Cl) at 2 h p.t. [56]. After 24 h, live cells were incubated with a mouse antibody against the HA tag (1:200), followed by incubation with an Alexa Fluor 568-conjugated antibody against mouse IgG for 60 min on ice. For intracellular staining of the M protein, cells were fixed with 4% PFA, permeabilized with 0.1% Triton X-100, and then incubated with 5% rabbit preimmune serum in PBS for 1 h. Then, NiV M was detected using the Zenon-labeled antibodies against the NiV M protein as described above.

### Incorporation of Br-UTP into nascent viral RNA

Cells grown on coverslips were infected with NiV at an MOI of 0.05. At 18 h p.i., medium was replaced with DMEM containing 2% FCS and supplemented with actinomycin D (10 μg/ml) to inhibit cellular transcription. After 1 h cells were transfected with 10 mM Br-UTP using Lipofectamine 2000, and were incubated in ActD containing medium for 20 or 60 min to allow Br-UTP incorporation into *de novo* synthesized viral RNA. The cells were then extensively washed with PBS and were fixed with 4% PFA. After removal from the BSL-4 lab, immunofluorescence staining was performed as described above.

### 5-ethynyl-uridine labeling

Vero76 and Huh-7 were grown on coverslips and infected with NiV or Ebola virus (EBOV isolate Mayinga, kindly provided by Stephan Becker) at MOI 0.05 and 0.01, respectively. At 18 h p.i., RNA staining was performed using the Click-iT nascent RNA detection kit following the manufacturer's instructions. Briefly, cellular transcription was blocked for 60 min by adding DMEM 2% FCS containing 10 μg ActD per ml. Then ActD-containing medium was supplemented with 5-ethynyl-uridine (EU; 2 mM). After EU incorporation into nascent RNA for 60 min, cells were washed five times with PBS and fixed with 4% PFA for 48 h. After removal from the BSL-4 facility, the samples were washed, permeabilized with 0.1% Triton X-100 for 15 min, washed again and then incubated with Alexa Fluor 488-azide in Click-iT buffer for 30 min at room temperature. Subsequently, IBs in NiV-infected cells were immunostained as described above. Inclusions in EBOV-infected cells were detected with a polyclonal goat serum directed against EBOV [57].

### FISH

To detect NiV mRNA in infected cells, a fluorescence in-situ hybridization (FISH) protocol previously described by Cifuentes-Munoz et al. [19] was adapted. Based on the full-length NiV_Malaysia_ sequence, a set of 48 Stellaris FISH probes complementary to (+) sense RNA (+RNA) covering the genes for N, P and M (nucleotides 1–6000) was generated by BioSearch Technologies (Novato, CA). The probes were designed with the software provided by the company. Before the purchase, the sequences of the probes were checked and any probes complementary to (-) sense vRNA were excluded. Each +RNA probe had a length of 20 nucleotides and was conjugated to a Quasar 670 fluorophore. For the *in situ* hybridization, Vero76 cells were infected with NiV. At 18 h p.i., cells were fixed with 4% PFA for 48 h and then removed from the BSL-4 facility. PFA was quenched with 0.1 M glycine for 15 min at room temperature, followed by washing with PBS twice. Cells were then permeabilized with 70% ethanol for 18 h at 4° C. Hybridization was performed strictly following the protocol of the manufacturer (Stellaris RNA FISH, Protocol for Adherent cells) using the wash and hybridization buffers provided by BioSearch Technologies. After hybridization overnight at 37° C, samples were washed and immunostaining of IBs was performed as described above, but the Triton X-100 permeabilization step was omitted. To control the specificity of the FISH probes, Vero76 cells were transfected with pCG plasmids encoding either NiV N, P or G protein. Cells were fixed with 4% PFA for 15 min and proceeded for FISH as described above. Confirming the specificity of the +RNA probes, only P and N mRNA but not G mRNA was detected in the transfected cells.

## Supporting information

S1 Fig IB^PM^ in NiV-infected cells visualized by TEM. Vero76 cells were infected with wildtype NiV at a MOI of 2. Infected cells were fixed and processed for transmission electron microscopy at 24 h p.i.. Ultrathin sections of two cells with multiple IB^PM^ are shown. The inner outlines of the IBs are indicated by white dotted lines and arrows. Scale bars, 1 μm.
IBs at the plasma membrane differ in sizes and shapes. Black arrows indicate IB^PM^ forming rather thin layers underneath the plasma membrane. White arrows point to larger IB^PM^ structures, one with an almost square shape. Independent on their overall form, IB^PM^ generally cover large areas of the plasma membrane, which explains why IBs appear relatively large and pleomorphic in the immunostainings, since they always show a top view of the cells.
(TIF)

S2 Fig Distribution of NCs in NiV-infected cells. Vero76 cells were infected with wildtype NiV at a MOI of 2. Infected cells were fixed and processed for transmission electron microscopy at 24 h p.i.. The dotted lines indicate an IB^PM^ and an IB_peri_. The bottom panels show enlarged views of NCs (arrows) in IB^PM^ (blue boxed area), IB_peri_ (green boxed area), and NC-like structures in the cytoplasm outside of IBs (red boxed area).
(TIF)

S3 Fig IB distribution in different optical sections in the NiV-induced syncytium shown in Fig 2A. To better illustrate the threedimensonal distribution of IBs in syncytia formed due the fusion of lateral plasma membranes of neighboring cells, we analyzed the N and M staining in multiple confocal top-to-bottom sections of the syncytium shown in Fig 2A.
**(A)** Individual and merged images of a top, a center and a bottom section are shown. Yellow IBs in the merged images indicate M-positive IBs (IB^PM^), while green IBs represent M-negative IBs (IB_peri_).
**(B)** A maximum projection of all z-stack sections is shown. The dotted line indicates the approximate lateral border of the syncytium. Scale bar, 10 μm.
IB_peri_ (M-negative IBs) were only found in central and bottom regions of the multinucleated syncytium, many of them located in the regions close to the nuclei. Contrasting IB_peri_, lots of IB^PM^ (yellow) were located close to the indicated lateral border of the syncytium. Some M-positive IBs (IB^PM^) however appear to be located in central regions of the syncytium, even partly overlaying the nuclei in the maximum projection (B). These “central” IB^PM^ were only seen in top sections of the syncytium (A, top panel) indicating that these are associated with plasma membrane regions that are located above the nuclei. Once formed, an IB^PM^ probably stays where it was formed, so it appears to be located in the center of a syncytium, when cell fusion progresses and the syncytium and thus its lateral borders expand.
(TIF)

S4 Fig IB formation in NiV-infected bat cells. EidNi/43.1 cells [50] were infected with wildtype NiV at a MOI of 0.01. At 24 h p.i., cells were fixed and permeabilized with Triton X-100. Immunostaining of NiV N (green) and M (red) was performed as described in the legend to Fig 2. Since IB_peri_ do not contain M protein they appear in green. IB^PM^ were N- and M-positive and therefore appear in yellow. Scale bar, 10 μm. Merged images of three representative cells are shown.
Both IB subpopulation could be readily detected in NiV-infected bat cells showing that the two IB subpopulations, we originally identified in Vero76 cells, were also formed in bat cells. While the moderately infected cells in (A) and (B) had formed smaller and larger IB_peri_ and some IB^PM^ at the plasma membranes, the heavily infected cell in (C) contained huge pleomorphic IB^PM^ covering almost the complete cell border. In this cell, IB_peri_ were rare, similar to what is observed in other cell types when many IB^PM^ have formed. This demonstrates that IB_peri_ and IB^PM^ formation is a common characteristic of NiV infection, even in cells that do not undergo rapid syncytium formation as do Vero76 cells.
(TIF)

S5 Fig Surface localization of NiV G glycoprotein in the presence and absence of IB^PM^. Vero76 cells were transfected to coexpress the NiV proteins F, G_HA_, N, and P_eGFP_ in the presence (A) or absence of the M protein (B). To facilitate the surface staining of the NiV glycoproteins, 20 mM NH_4_Cl was added to inhibit cell-cell fusion [56]. 24 h after transfection, live cells were surface-labeled with an anti-HA antibody on ice (red). After G staining, cells were fixed with 4% PFA and permeabilized with 0.1% Triton X-100, followed by incubation with a Zenon-labeled anti-M peptide serum (cyan). IBs were detected by P_eGFP_ autofluorescence (green). Nuclei were stained with DAPI (blue). Scale bars, 10 μm.
Panel (A) shows that surface-expressed NiV G proteins clearly colocalized with the M protein in IB^PM^. In the absence of the M protein (panel B), IB^PM^ were not formed and surface glycoproteins were homogenously distributed on the plasma membrane.
(TIF)

S6 Fig IB formation in Huh-7 cells in the absence and presence of NiV M. NiV N and NiV P_eGFP_ proteins were coexpressed in a human hepatoma cell line (Huh-7) either alone (A) or together with the NiV M protein (B). 24 h after transfection, cells were fixed, permeabilized with 0.1% Triton X-100 and immunostained as described in the legend to Fig 3. Scale bars, 10 μm.
Confirming the observation in Vero76 cells (Fig 3), N and P protein expressed in Huh-7 cells (panel A) resulted in the formation of IBs which are mostly round and located in the perinuclear region (IB_peri_). Upon coexpression of the M protein (panel B), mostly larger, pleomorphic shaped M-positive IBs in close vicinity to the plasma membrane were found (IB^PM^), while M-negative IB_peri_ were much less abundant.
(TIF)

S7 Fig IBs in NiV-infected cells at early and late time points p.i.. Vero76 cells were infected with wildtype NiV at a MOI of 0.05. At 5 h and 18 h p.i. cells were fixed with 4% PFA for 48 h and permeabilized with Triton X-100. IBs (N protein, green) and NiV M (pseudo-coloured in red) were immunostained as described in the legend to Fig 5. Scale bars, 10 μm.
Confirming the observations in NiV-infected cells at 24 h p.i. (Fig 2A), M-positive IBs in peripheral membrane-proximal regions (IB^PM^) could be detected at 18 h p.i.. At 5 h p.i. when the NiV M protein was not yet expressed at detectable levels, only IBs in the perinuclear region (IB_peri_) were formed. This clearly supports the idea that the kinetics of IB_peri_ and IB^PM^ formation differ. Consistent with the live cell imaging showing IB formation in transfected cells (S1 Movie), N-positive IB_peri_ were present in infected cells at very early time points, while N- and M-positive IB^PM^ were only detected later when sufficient M protein was expressed.
(TIF)

S8 Fig Localization of Br-UTP labeled viral RNA and eIF4G in NiV-infected cells. Vero76 cells were infected with NiV at a MOI of 0.05. At 18 h p.i. cells were treated for 1 h with actinomycin D to inhibit cellular transcription or left untreated. Then, cells were transfected with 10 mM Br-UTP. After RNA labelling for 60 min, cells were fixed and permeabilized with methanol/acetone. Viral RNAs were detected using a Br-UTP monoclonal antibody and AF568-labeled anti-mouse antibodies (red). After blocking with mouse serum, eIF4G was detected with specific mouse antibodies and AF647-labeled secondary antibodies (cyan). Then IBs were visualized with an NiV N-specific antiserum and AF488-labeled secondary antibodies (green). Nuclei were counterstained with DAPI (blue).
**(A)** Cellular RNA staining in uninfected control cells (Mock) without (-ActD) and with inhibitor (+ActD) are shown.
**(B)** Colocalization of viral RNA, eIF4G and IBs in actinomycin D-treated NiV-infected cells. In the zoom panel, enlarged views of IB_peri_ and IB^PM^ are shown. Arrows indicate RNA dots. Scale bars, 10 μm.
As also shown in Fig 6B, Br-UTP labeled viral RNA showed a punctuate staining pattern and did not substantially colocalize with IBs. The RNA dots were detected throughout the cytoplasm and colocalized with the mRNA binding protein eIF4G indicating that the Br-UTP labeled RNA mostly presents viral mRNA rather than genomic or antigenomic RNA.
(TIF)

S9 Fig Colocalization of EU-labeled *de novo* synthesized viral RNA and IBs. Vero76 and Huh-7 cells were infected with NiV (C) and EBOV (D) at MOI 0.05 and 0.01, respectively. At 18 h p.i., actinomycin D (ActD) was added to the cells to inhibit cellular RNA synthesis. 60 min later, ethynyl-uridine (EU) was added to the medium for 1 h before cells were fixed and permeabilized with Triton X-100. EU incorporated into nascent RNAs was detected using AF488-azide.
**(A, B)** Cellular RNA staining in uninfected control Vero76 (A) and Huh-7 cells (B) without (-ActD) and with inhibitor (+ActD) are shown.
**(C)** NiV N (red) and M proteins (cyan) in NiV IBs were immunostained with an NiV N-specific antiserum and Zenon-labeled anti-M peptide serum
**(D)** IBs (red) in EBOV-infected cells were visualized with an anti-EBOV goat serum [57]. Nuclei were counterstained with DAPI. Confocal sections of the infected cells are shown. Scale bars, 10 μm.
As shown before by Hoenen et al. [25], EBOV RNA could be colocalized with EBOV inclusions (D). In contrast, no EU-labeled RNA could be specifically detected in NiV-infected cells (C). This supports the conclusions drawn from the Br-UTP labeling (Figs 6 and S8) that NiV RNA synthesis does not take place in IBs.
(TIF)

S10 Fig Colocalization of NiV IB_peri_ and cellular compartment markers. Vero76 cells were transfected with plasmids encoding NiV N and NiV P_eGFP_ to form IB_peri_. At 24 h p.t., cells were fixed, permeabilized with Triton X-100 and immunostained with antibodies directed against cellular marker proteins. In the right panel (zoom IB_peri_), enlarged views of the merged confocal images are shown. Scale bars, 10 μm.
Panel (A) shows that IB_peri_ did not colocalize with EEA1 (early endosome), Lamp1 (late endosome or lysosome), Calnexin (ER), or GM130 (Golgi). As shown in panel (B), the stress granule marker G3BP1 was not recruited to IB_peri._
(TIF)

S11 Fig Colocalization of NiV IB_peri_ with y-tubulin and mCherry in Huh-7 cells. Huh-7 cells were transfected with NiV N and NiV P_eGFP_ to form IBs (IB_peri_). At 24 h p.t. cells were fixed with 4% PFA and permeabilized with methanol/acetone. IBs were detected by P_eGFP_ autofluorescence (IB_peri_).
**(A)** Cellular aggresome marker γ-tubulin was detected with specific antibodies (red).
**(B)** NiV N and NiV P_eGFP_ were coexpressed with the non-related cytosolic reporter mCherry protein (red) which was detected by autofluorescence. Nuclei were counterstained with DAPI (blue). Only merged confocal images are shown. IBs within the boxed areas are shown at higher magnification. Scale Bars, 10 μm.
Colocalization of NiV IB_peri_ with y-tubulin in Huh-7 cells and recruitment of mCherry confirms the aggresome-like character of IB_peri_ observed in Vero76 cells (Fig 7).
(TIF)

S12 Fig Colocalization of NiV IB^PM^ with y-tubulin and mCherry. Vero76 cells were transfected with NiV N, NiV P_eGFP_ and NiV M to form IBs (IB^PM^). At 24 h p.t. cells were fixed with 4% PFA and permeabilized with methanol/acetone. IBs were detected by P_eGFP_ autofluorescence (green) and by M immunostaining using a Zenon-labeled anti-M peptide serum (cyan).
**(A)** Cellular aggresome marker γ-tubulin was detected with specific antibodies (red).
**(B)** NiV N, P_eGFP_ and M were coexpressed with the non-related cytosolic reporter mCherry protein (red) which was detected by autofluorescence. Nuclei were counterstained with DAPI (blue). Scale Bars, 10 μm.Magnifications of the confocal images and individual staining of the boxed areas are presented in the bottom panel.
The lack of y-tubulin and mCherry in IB^PM^ shows that they differ from IB_peri_ by neither recruiting cellular aggresome markers nor unrelated cytosolic proteins.
(TIF)

S1 Movie Formation NiV IB_peri_ and IB^PM^ monitored by live cell imaging. To follow IB formation in the presence of M over time, Vero76 cells were cotransfected with NiV N and NiV P_eGFP_ together with _mCherry_NiV M and untagged NiV M. At 14 h p.t., the live cell time-lapse experiments were started. Images were recorded with a Nikon TE2000 microscope. Pictures were taken every 50 sec. Scale bar, 10 μm.
The video shows that round IBs are formed in the cytoplasm. These IB_peri_ (green) do not contain M protein and constantly move within the cytoplasm. IB^PM^ (yellow) are only formed later at the plasma membrane and contain M protein from the initial stages of formation.
(MP4)

S2 Movie Live cell imaging of IB formation in the absence of NiV M protein. For comparison, IB formation was followed in the absence of the NiV M protein. For this, Vero76 cells were cotransfected with only NiV N and NiV P_eGFP_.
The video again demonstrates that cytoplasmic IBs (IB_peri_) are highly mobile within the cytoplasm and can fuse with each other. There is no formation or accumulation of IBs at the plasma membrane. Scale bar, 10 μm.
(MP4)
