# Supplementary figures and images for: Nipah virus induces two inclusion body populations: Identification of novel inclusions at the plasma membrane
Source: PLoS Pathog. 2019 Apr 29;15(4):e1007733. doi: 10.1371/journal.ppat.1007733 (PMC6488097; doi:10.1371/journal.ppat.1007733)

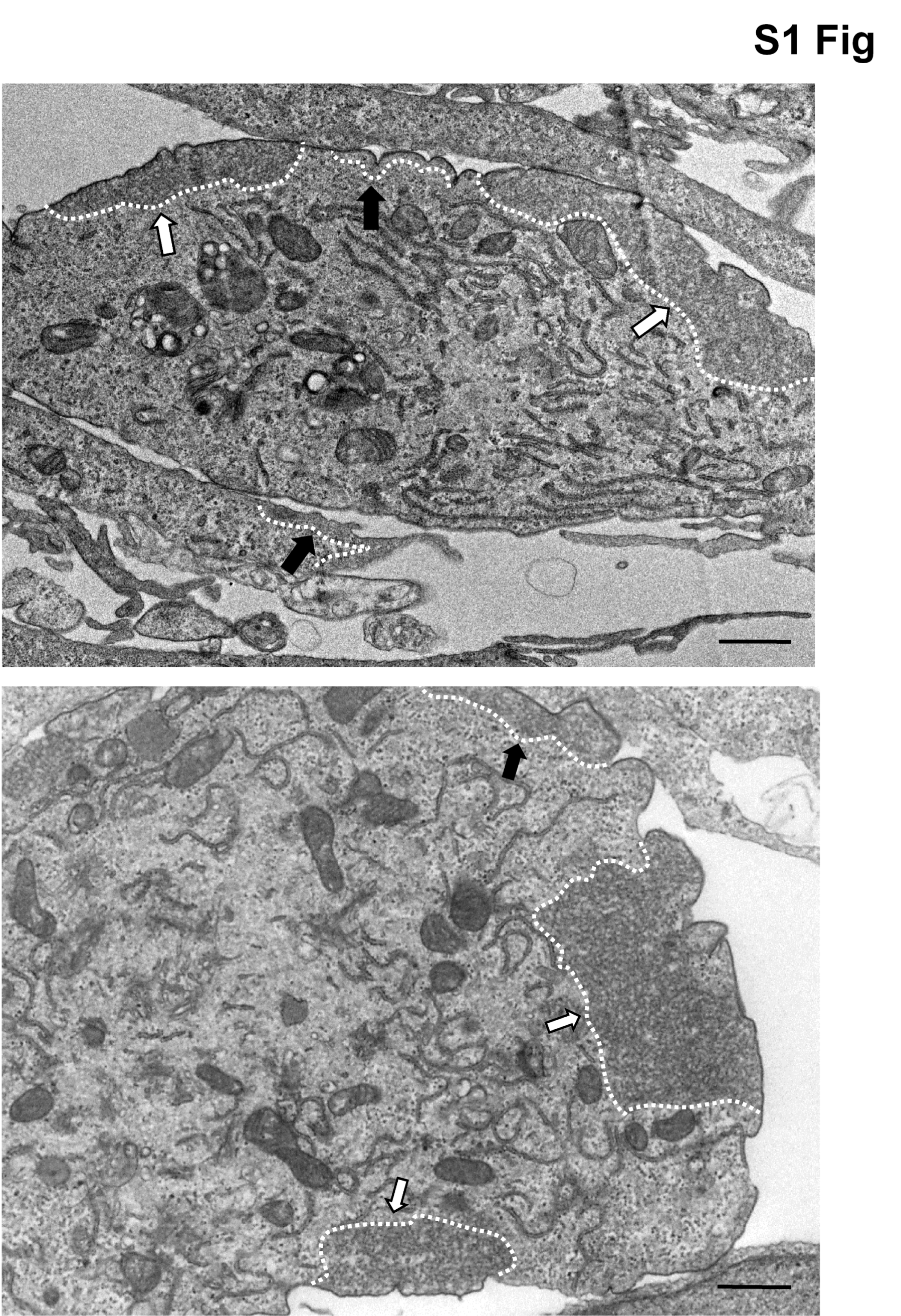

Supplement: S1 Fig — Vero76 cells were infected with wildtype NiV at a MOI of 2. Infected cells were fixed and processed for transmission electron microscopy at 24 h p.i.. Ultrathin sections of two cells with multiple IBPM are shown. The inner outlines of the IBs are indicated by white dotted lines and arrows. Scale bars, 1 μm. IBs at the plasma membrane differ in sizes and shapes. Black arrows indicate IBPM forming rather thin layers underneath the plasma membrane. White arrows point to larger IBPM structures, one with an almost square shape. Independent on their overall form, IBPM generally cover large areas of the plasma membrane, which explains why IBs appear relatively large and pleomorphic in the immunostainings, since they always show a top view of the cells. (TIF) [file ppat.1007733.s001.tif]

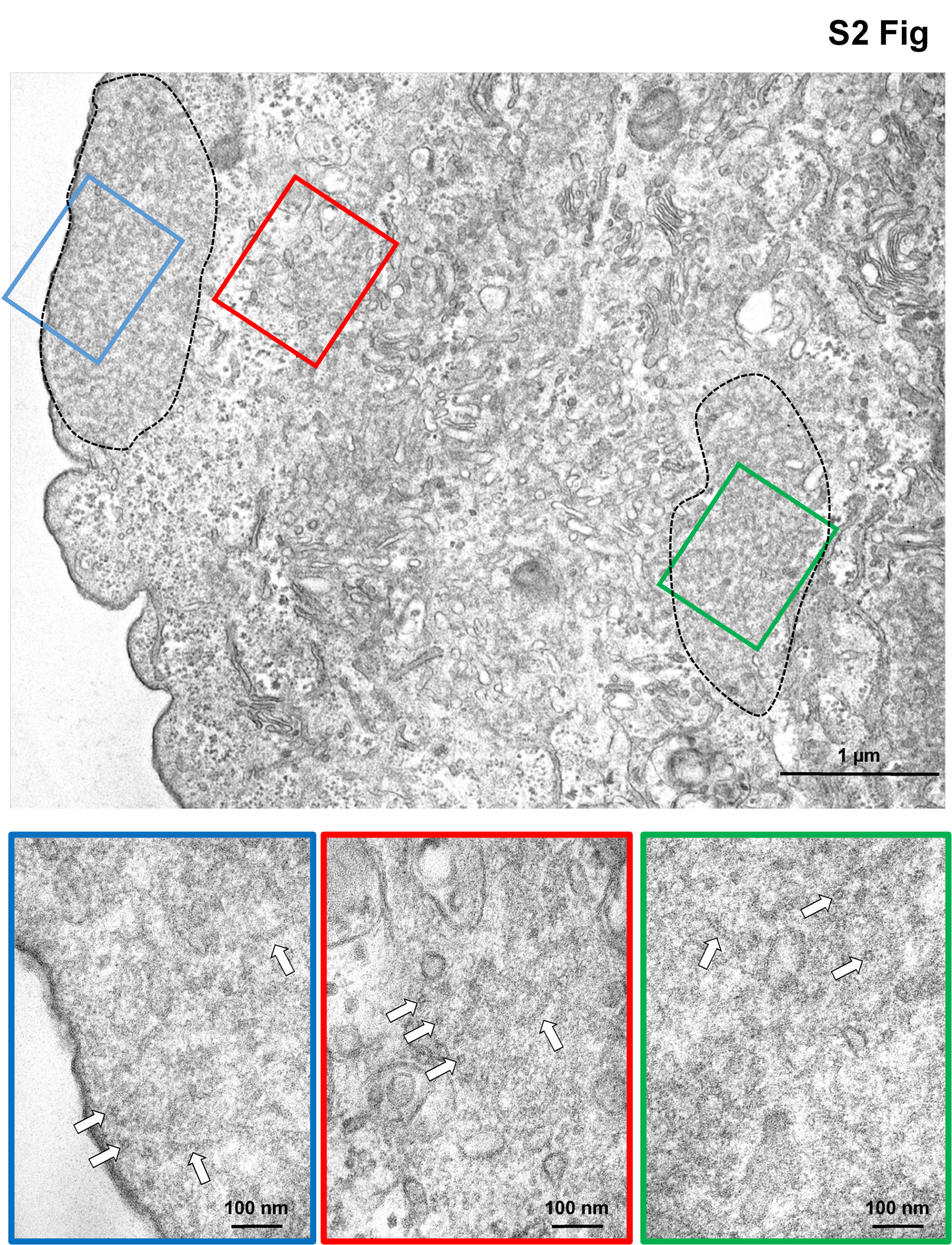

Supplement: S2 Fig — Vero76 cells were infected with wildtype NiV at a MOI of 2. Infected cells were fixed and processed for transmission electron microscopy at 24 h p.i.. The dotted lines indicate an IBPM and an IBperi. The bottom panels show enlarged views of NCs (arrows) in IBPM (blue boxed area), IBperi (green boxed area), and NC-like structures in the cytoplasm outside of IBs (red boxed area). (TIF) [file ppat.1007733.s002.tif]

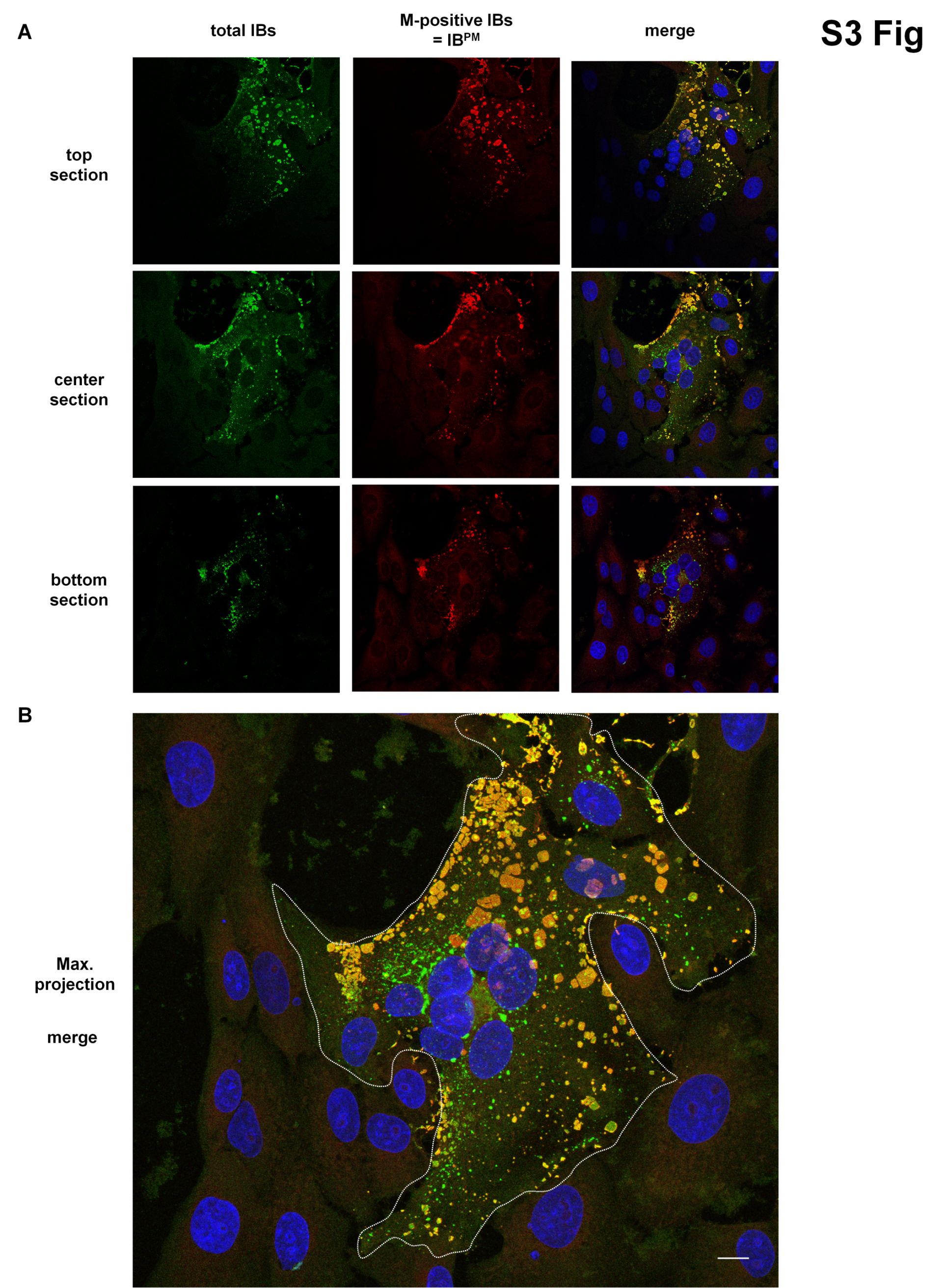

Supplement: S3 Fig — To better illustrate the threedimensonal distribution of IBs in syncytia formed due the fusion of lateral plasma membranes of neighboring cells, we analyzed the N and M staining in multiple confocal top-to-bottom sections of the syncytium shown in Fig 2A. (A) Individual and merged images of a top, a center and a bottom section are shown. Yellow IBs in the merged images indicate M-positive IBs (IBPM), while green IBs represent M-negative IBs (IBperi). (B) A maximum projection of all z-stack sections is shown. The dotted line indicates the approximate lateral border of the syncytium. Scale bar, 10 μm. IBperi (M-negative IBs) were only found in central and bottom regions of the multinucleated syncytium, many of them located in the regions close to the nuclei. Contrasting IBperi, lots of IBPM (yellow) were located close to the indicated lateral border of the syncytium. Some M-positive IBs (IBPM) however appear to be located in central regions of the syncytium, even partly overlaying the nuclei in the maximum projection (B). These “central” IBPM were only seen in top sections of the syncytium (A, top panel) indicating that these are associated with plasma membrane regions that are located above the nuclei. Once formed, an IBPM probably stays where it was formed, so it appears to be located in the center of a syncytium, when cell fusion progresses and the syncytium and thus its lateral borders expand. (TIF) [file ppat.1007733.s003.tif]

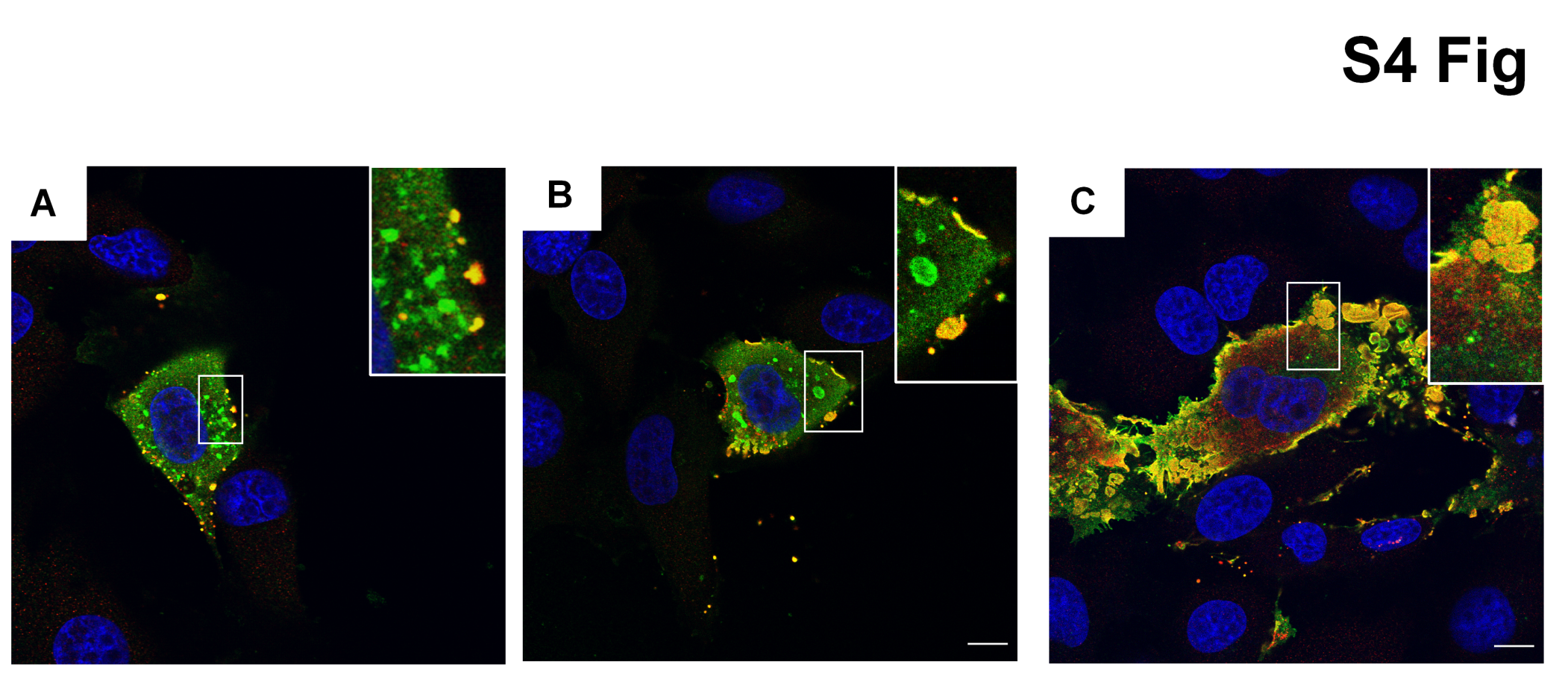

Supplement: S4 Fig — EidNi/43.1 cells [50] were infected with wildtype NiV at a MOI of 0.01. At 24 h p.i., cells were fixed and permeabilized with Triton X-100. Immunostaining of NiV N (green) and M (red) was performed as described in the legend to Fig 2. Since IBperi do not contain M protein they appear in green. IBPM were N- and M-positive and therefore appear in yellow. Scale bar, 10 μm. Merged images of three representative cells are shown. Both IB subpopulation could be readily detected in NiV-infected bat cells showing that the two IB subpopulations, we originally identified in Vero76 cells, were also formed in bat cells. While the moderately infected cells in (A) and (B) had formed smaller and larger IBperi and some IBPM at the plasma membranes, the heavily infected cell in (C) contained huge pleomorphic IBPM covering almost the complete cell border. In this cell, IBperi were rare, similar to what is observed in other cell types when many IBPM have formed. This demonstrates that IBperi and IBPM formation is a common characteristic of NiV infection, even in cells that do not undergo rapid syncytium formation as do Vero76 cells. (TIF) [file ppat.1007733.s004.tif]

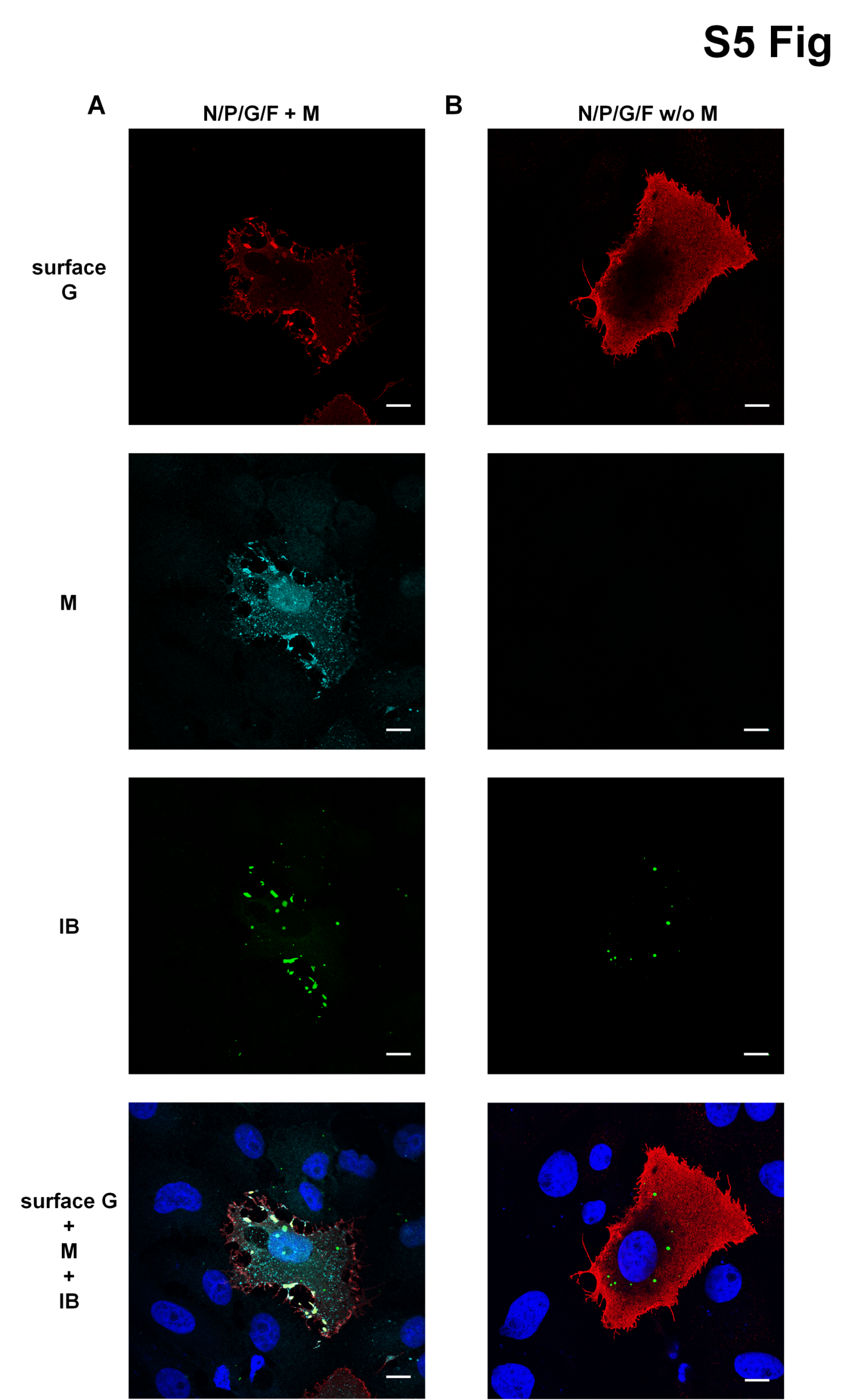

Supplement: S5 Fig — Vero76 cells were transfected to coexpress the NiV proteins F, GHA, N, and PeGFP in the presence (A) or absence of the M protein (B). To facilitate the surface staining of the NiV glycoproteins, 20 mM NH4Cl was added to inhibit cell-cell fusion [56]. 24 h after transfection, live cells were surface-labeled with an anti-HA antibody on ice (red). After G staining, cells were fixed with 4% PFA and permeabilized with 0.1% Triton X-100, followed by incubation with a Zenon-labeled anti-M peptide serum (cyan). IBs were detected by PeGFP autofluorescence (green). Nuclei were stained with DAPI (blue). Scale bars, 10 μm. Panel (A) shows that surface-expressed NiV G proteins clearly colocalized with the M protein in IBPM. In the absence of the M protein (panel B), IBPM were not formed and surface glycoproteins were homogenously distributed on the plasma membrane. (TIF) [file ppat.1007733.s005.tif]

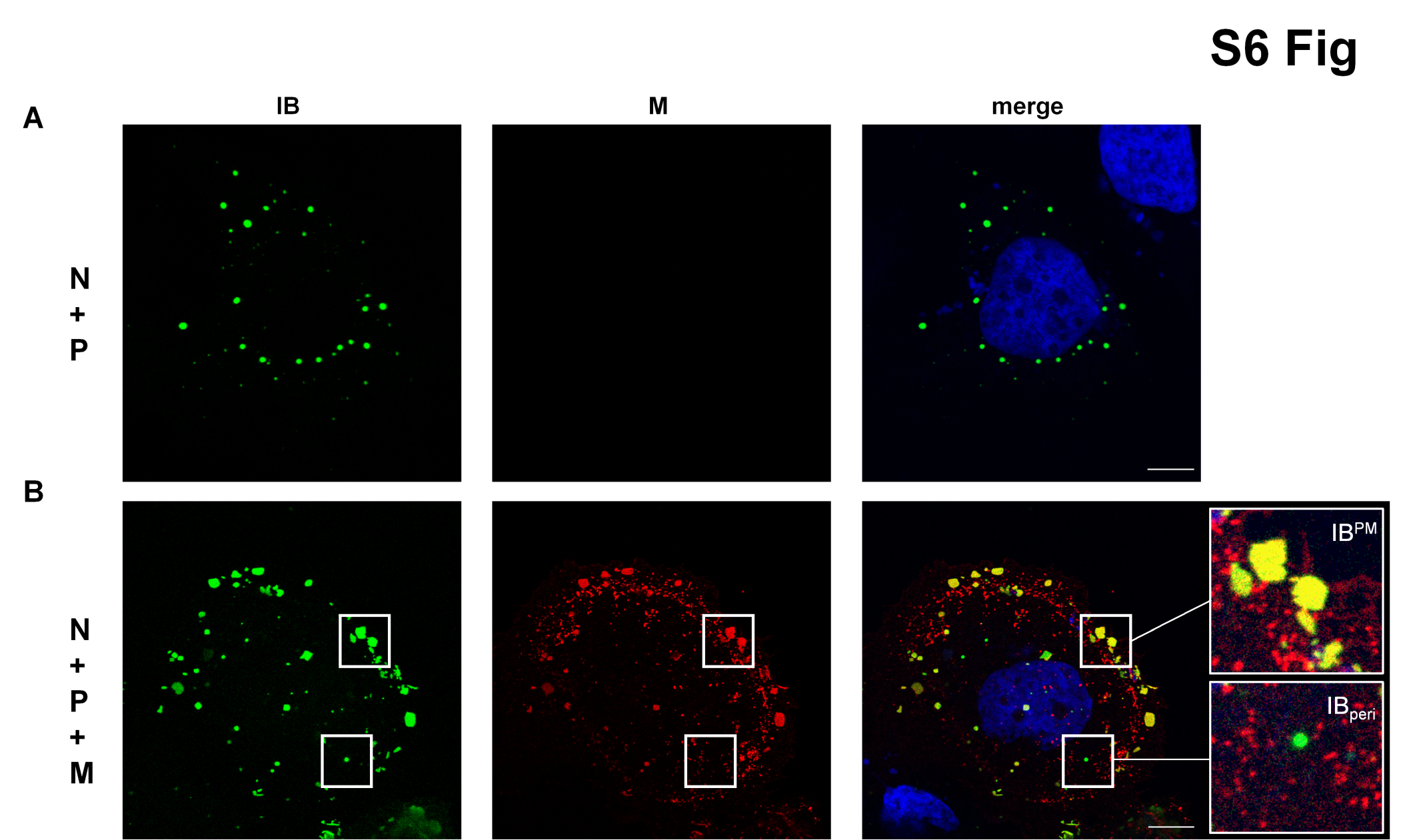

Supplement: S6 Fig — NiV N and NiV PeGFP proteins were coexpressed in a human hepatoma cell line (Huh-7) either alone (A) or together with the NiV M protein (B). 24 h after transfection, cells were fixed, permeabilized with 0.1% Triton X-100 and immunostained as described in the legend to Fig 3. Scale bars, 10 μm. Confirming the observation in Vero76 cells (Fig 3), N and P protein expressed in Huh-7 cells (panel A) resulted in the formation of IBs which are mostly round and located in the perinuclear region (IBperi). Upon coexpression of the M protein (panel B), mostly larger, pleomorphic shaped M-positive IBs in close vicinity to the plasma membrane were found (IBPM), while M-negative IBperi were much less abundant. (TIF) [file ppat.1007733.s006.tif]

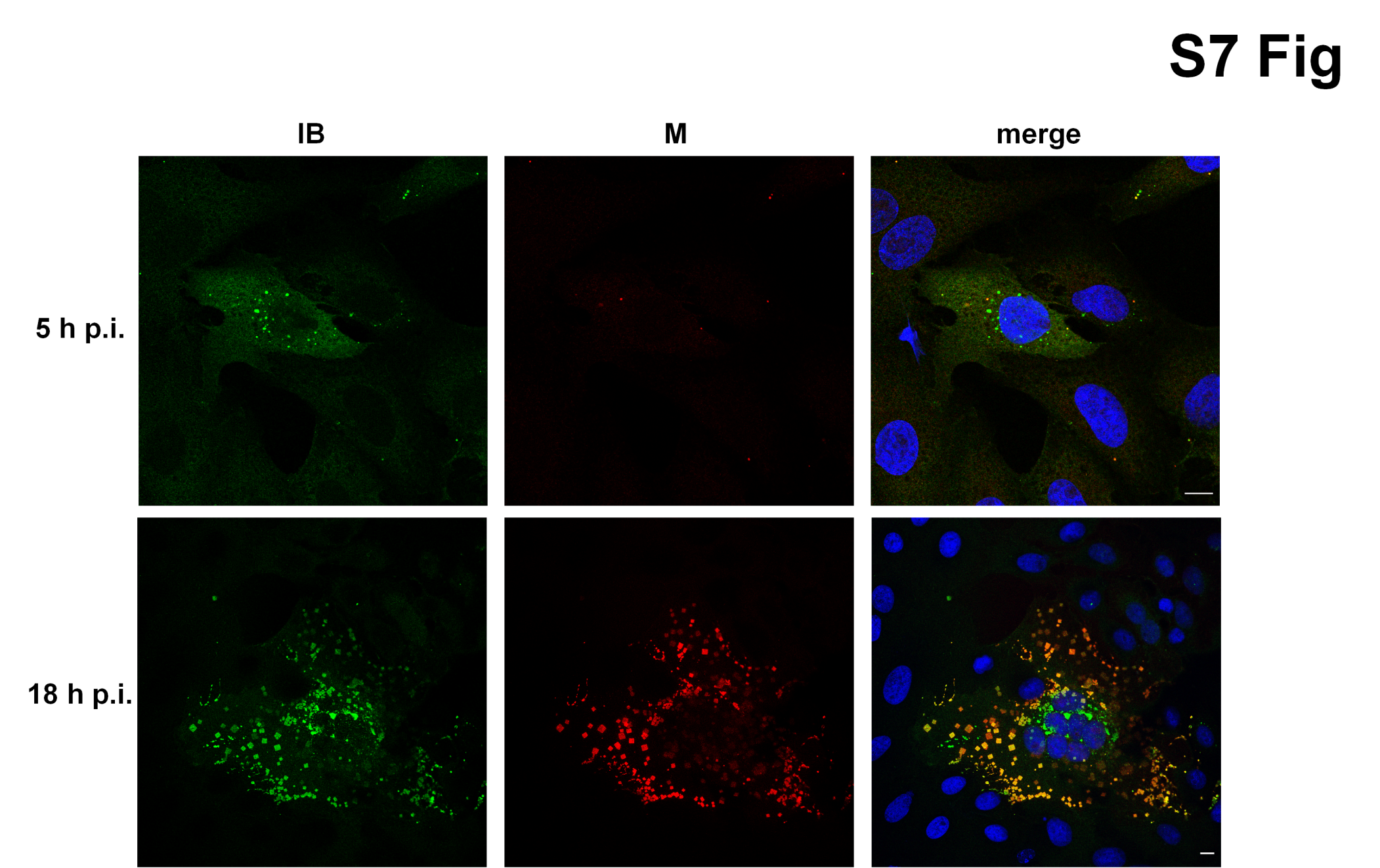

Supplement: S7 Fig — Vero76 cells were infected with wildtype NiV at a MOI of 0.05. At 5 h and 18 h p.i. cells were fixed with 4% PFA for 48 h and permeabilized with Triton X-100. IBs (N protein, green) and NiV M (pseudo-coloured in red) were immunostained as described in the legend to Fig 5. Scale bars, 10 μm. Confirming the observations in NiV-infected cells at 24 h p.i. (Fig 2A), M-positive IBs in peripheral membrane-proximal regions (IBPM) could be detected at 18 h p.i.. At 5 h p.i. when the NiV M protein was not yet expressed at detectable levels, only IBs in the perinuclear region (IBperi) were formed. This clearly supports the idea that the kinetics of IBperi and IBPM formation differ. Consistent with the live cell imaging showing IB formation in transfected cells (S1 Movie), N-positive IBperi were present in infected cells at very early time points, while N- and M-positive IBPM were only detected later when sufficient M protein was expressed. (TIF) [file ppat.1007733.s007.tif]

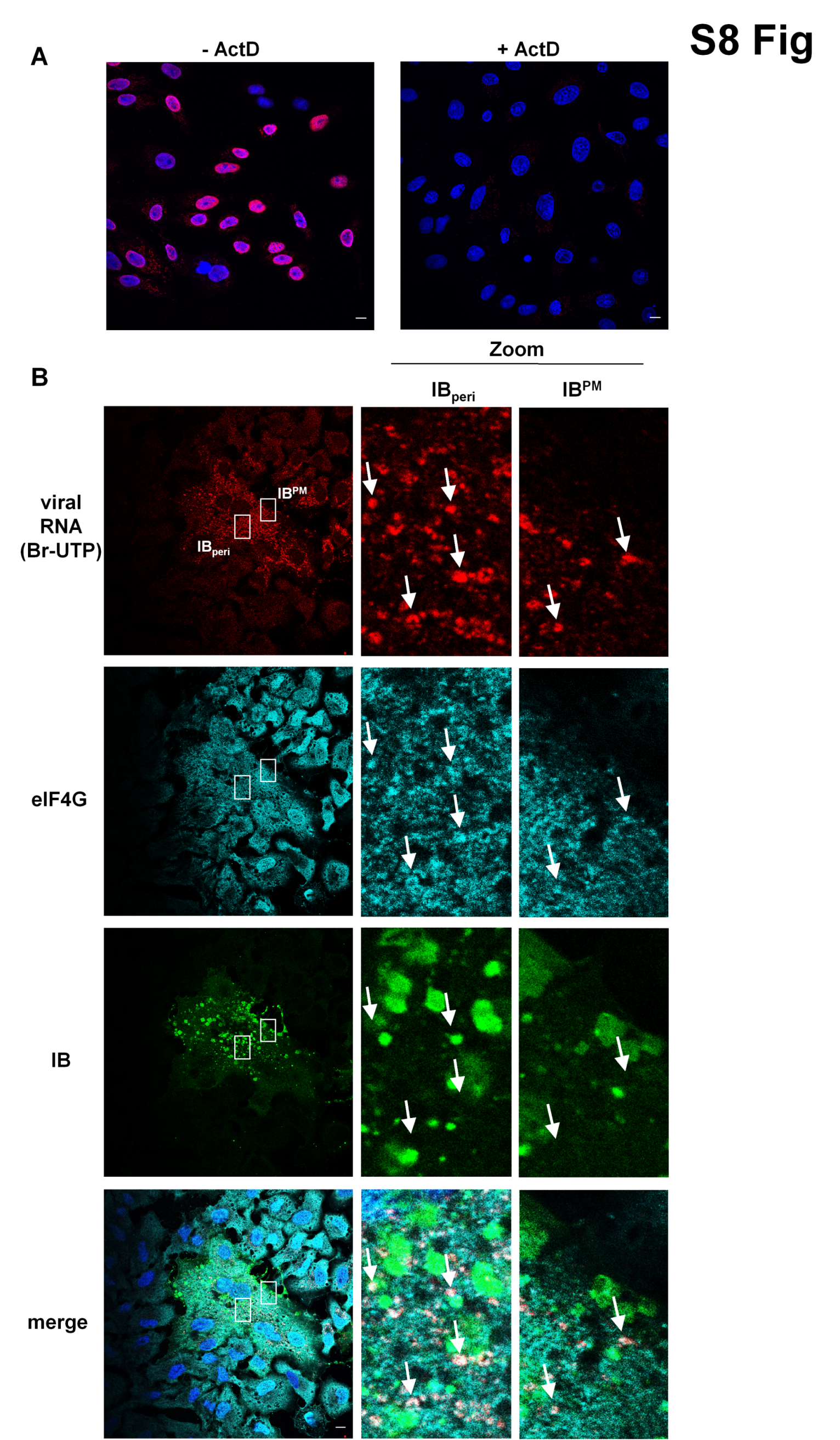

Supplement: S8 Fig — Vero76 cells were infected with NiV at a MOI of 0.05. At 18 h p.i. cells were treated for 1 h with actinomycin D to inhibit cellular transcription or left untreated. Then, cells were transfected with 10 mM Br-UTP. After RNA labelling for 60 min, cells were fixed and permeabilized with methanol/acetone. Viral RNAs were detected using a Br-UTP monoclonal antibody and AF568-labeled anti-mouse antibodies (red). After blocking with mouse serum, eIF4G was detected with specific mouse antibodies and AF647-labeled secondary antibodies (cyan). Then IBs were visualized with an NiV N-specific antiserum and AF488-labeled secondary antibodies (green). Nuclei were counterstained with DAPI (blue). (A) Cellular RNA staining in uninfected control cells (Mock) without (-ActD) and with inhibitor (+ActD) are shown. (B) Colocalization of viral RNA, eIF4G and IBs in actinomycin D-treated NiV-infected cells. In the zoom panel, enlarged views of IBperi and IBPM are shown. Arrows indicate RNA dots. Scale bars, 10 μm. As also shown in Fig 6B, Br-UTP labeled viral RNA showed a punctuate staining pattern and did not substantially colocalize with IBs. The RNA dots were detected throughout the cytoplasm and colocalized with the mRNA binding protein eIF4G indicating that the Br-UTP labeled RNA mostly presents viral mRNA rather than genomic or antigenomic RNA. (TIF) [file ppat.1007733.s008.tif]

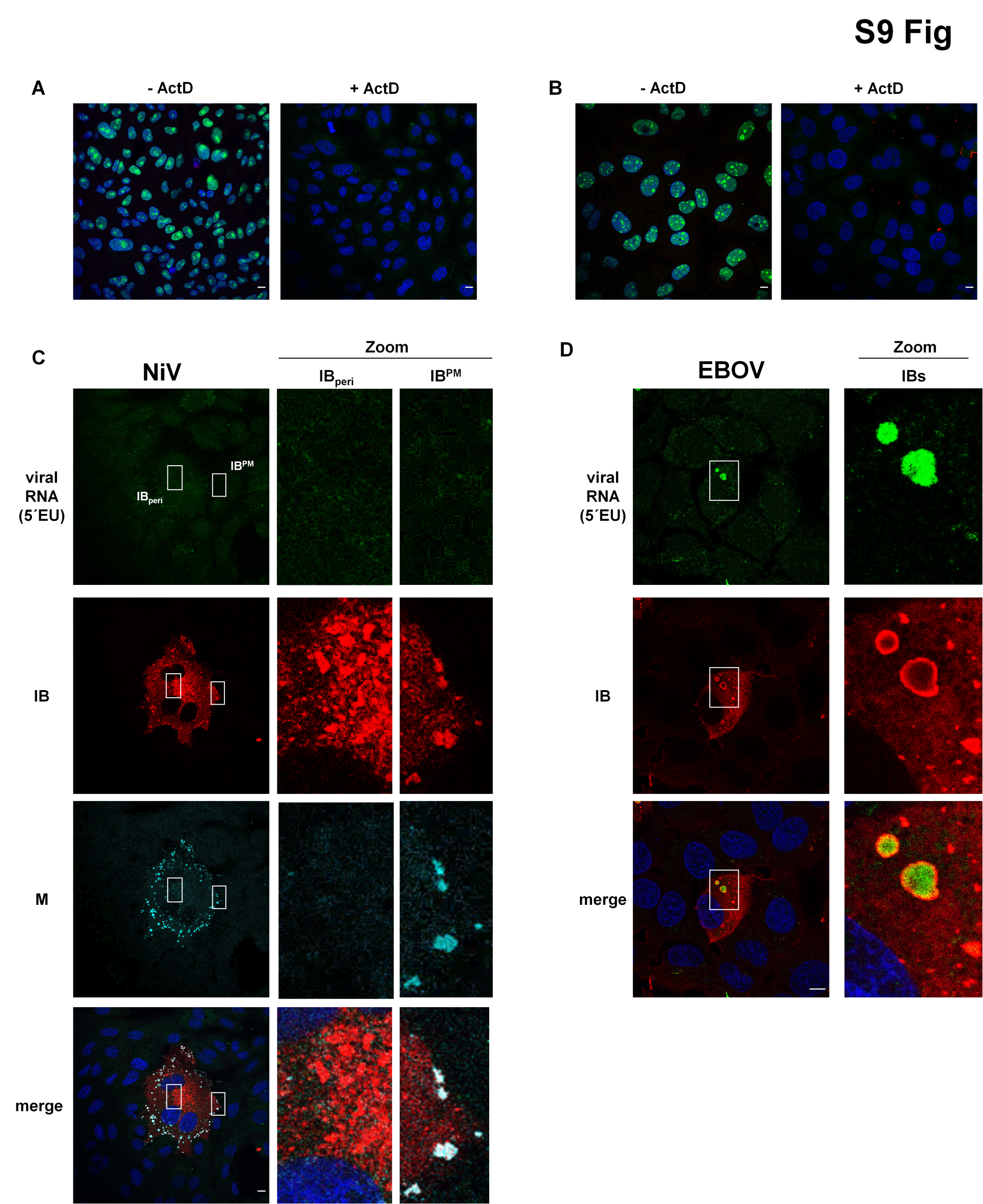

Supplement: S9 Fig — Vero76 and Huh-7 cells were infected with NiV (C) and EBOV (D) at MOI 0.05 and 0.01, respectively. At 18 h p.i., actinomycin D (ActD) was added to the cells to inhibit cellular RNA synthesis. 60 min later, ethynyl-uridine (EU) was added to the medium for 1 h before cells were fixed and permeabilized with Triton X-100. EU incorporated into nascent RNAs was detected using AF488-azide. (A, B) Cellular RNA staining in uninfected control Vero76 (A) and Huh-7 cells (B) without (-ActD) and with inhibitor (+ActD) are shown. (C) NiV N (red) and M proteins (cyan) in NiV IBs were immunostained with an NiV N-specific antiserum and Zenon-labeled anti-M peptide serum (D) IBs (red) in EBOV-infected cells were visualized with an anti-EBOV goat serum [57]. Nuclei were counterstained with DAPI. Confocal sections of the infected cells are shown. Scale bars, 10 μm. As shown before by Hoenen et al. [25], EBOV RNA could be colocalized with EBOV inclusions (D). In contrast, no EU-labeled RNA could be specifically detected in NiV-infected cells (C). This supports the conclusions drawn from the Br-UTP labeling (Figs 6 and S8) that NiV RNA synthesis does not take place in IBs. (TIF) [file ppat.1007733.s009.tif]

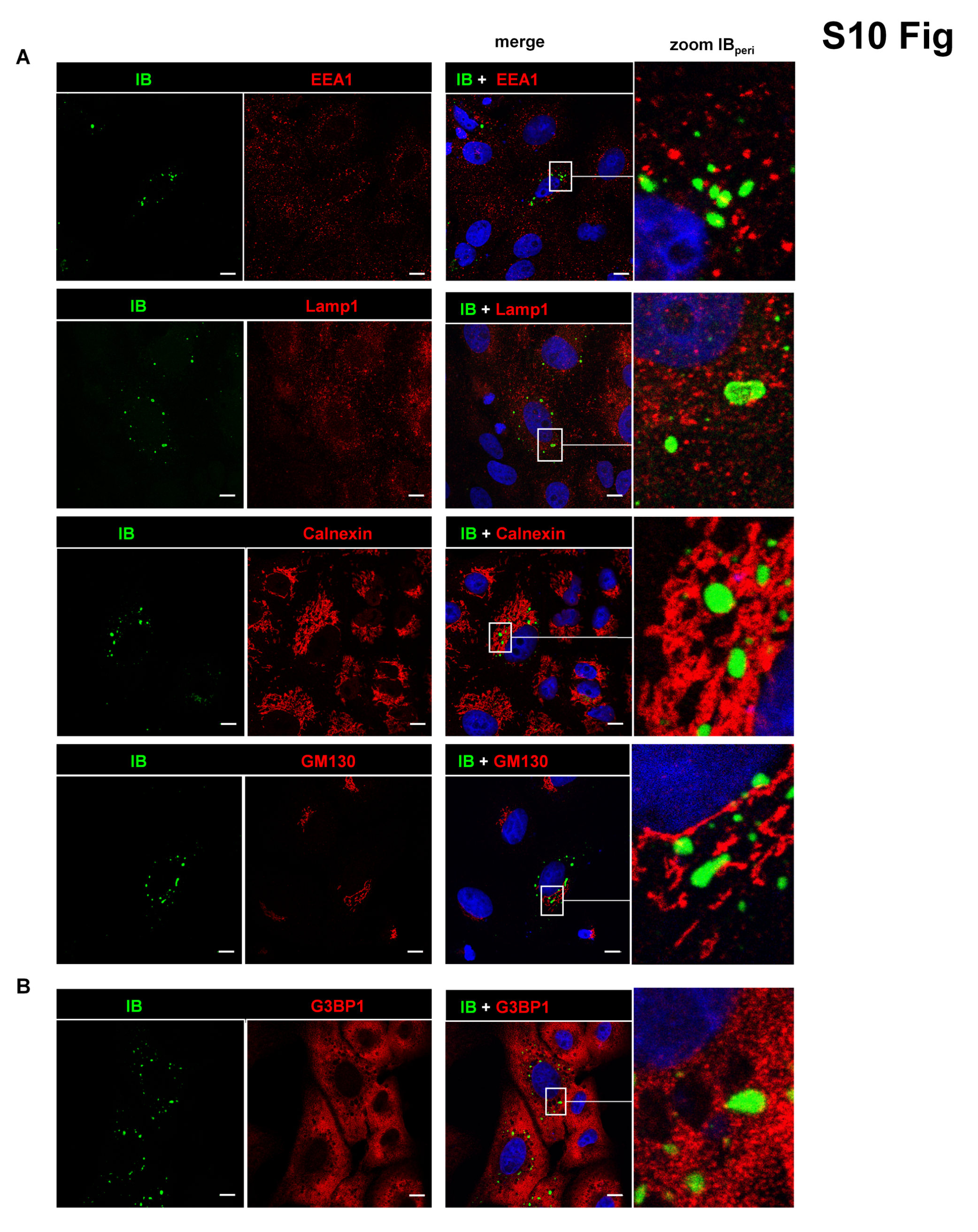

Supplement: S10 Fig — Vero76 cells were transfected with plasmids encoding NiV N and NiV PeGFP to form IBperi. At 24 h p.t., cells were fixed, permeabilized with Triton X-100 and immunostained with antibodies directed against cellular marker proteins. In the right panel (zoom IBperi), enlarged views of the merged confocal images are shown. Scale bars, 10 μm. Panel (A) shows that IBperi did not colocalize with EEA1 (early endosome), Lamp1 (late endosome or lysosome), Calnexin (ER), or GM130 (Golgi). As shown in panel (B), the stress granule marker G3BP1 was not recruited to IBperi. (TIF) [file ppat.1007733.s010.tif]

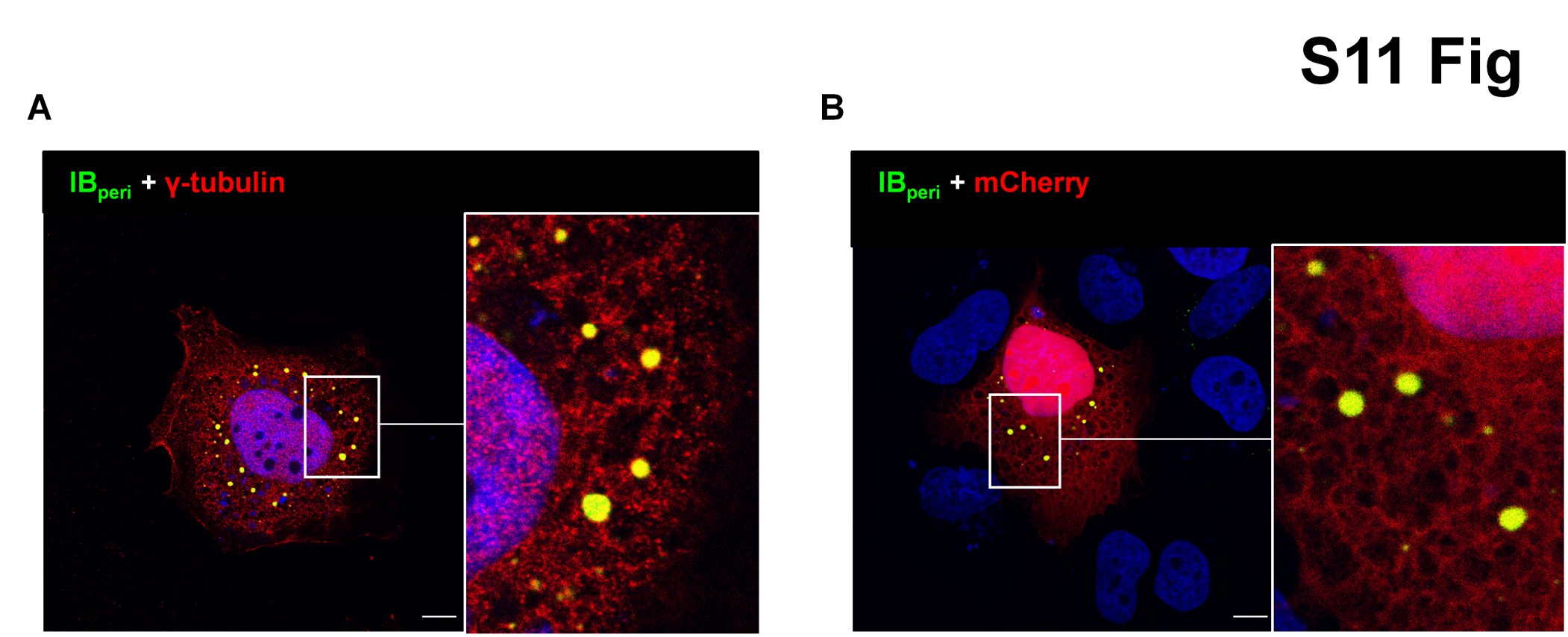

Supplement: S11 Fig — Huh-7 cells were transfected with NiV N and NiV PeGFP to form IBs (IBperi). At 24 h p.t. cells were fixed with 4% PFA and permeabilized with methanol/acetone. IBs were detected by PeGFP autofluorescence (IBperi). (A) Cellular aggresome marker γ-tubulin was detected with specific antibodies (red). (B) NiV N and NiV PeGFP were coexpressed with the non-related cytosolic reporter mCherry protein (red) which was detected by autofluorescence. Nuclei were counterstained with DAPI (blue). Only merged confocal images are shown. IBs within the boxed areas are shown at higher magnification. Scale Bars, 10 μm. Colocalization of NiV IBperi with y-tubulin in Huh-7 cells and recruitment of mCherry confirms the aggresome-like character of IBperi observed in Vero76 cells (Fig 7). (TIF) [file ppat.1007733.s011.tif]

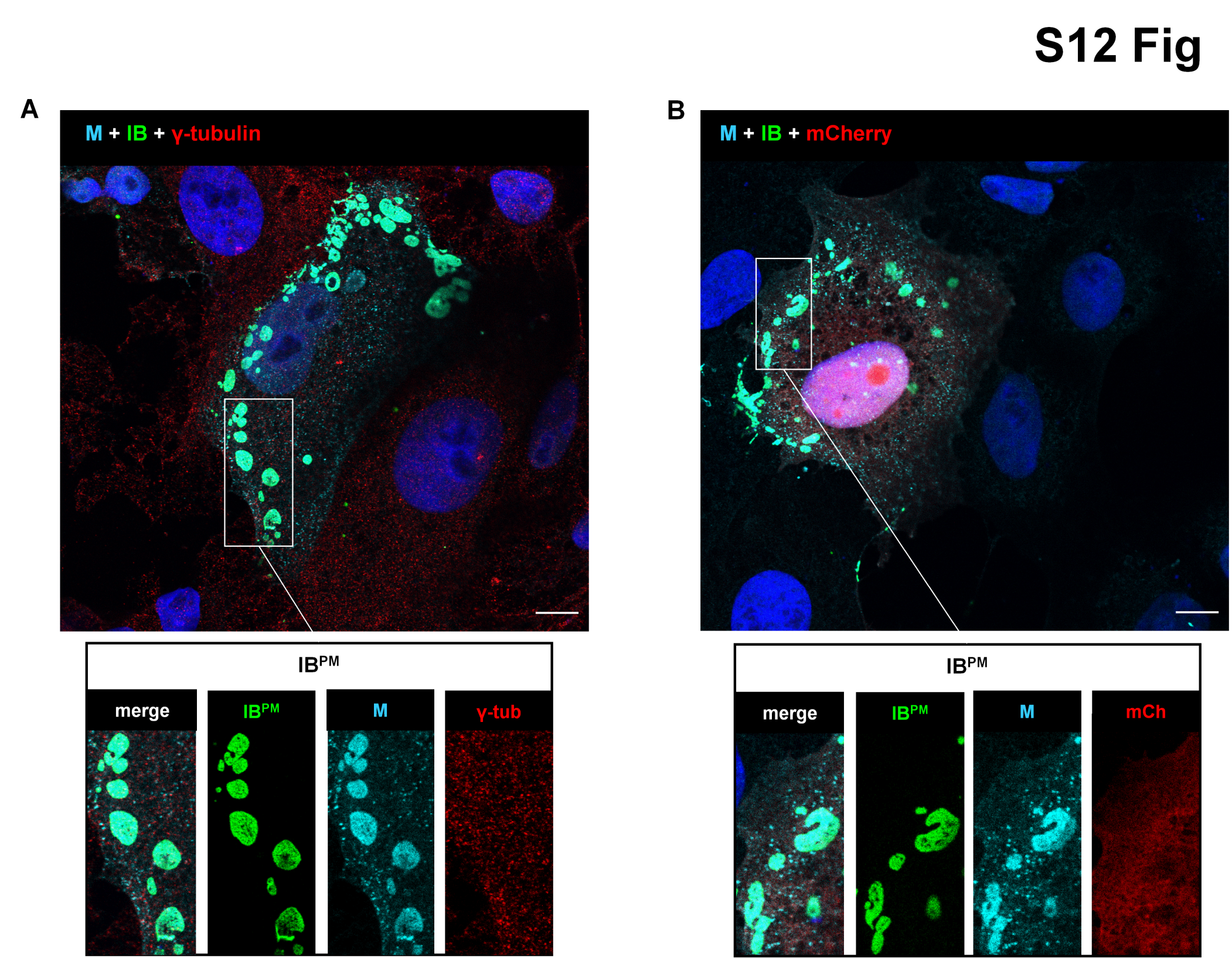

Supplement: S12 Fig — Vero76 cells were transfected with NiV N, NiV PeGFP and NiV M to form IBs (IBPM). At 24 h p.t. cells were fixed with 4% PFA and permeabilized with methanol/acetone. IBs were detected by PeGFP autofluorescence (green) and by M immunostaining using a Zenon-labeled anti-M peptide serum (cyan). (A) Cellular aggresome marker γ-tubulin was detected with specific antibodies (red). (B) NiV N, PeGFP and M were coexpressed with the non-related cytosolic reporter mCherry protein (red) which was detected by autofluorescence. Nuclei were counterstained with DAPI (blue). Scale Bars, 10 μm.Magnifications of the confocal images and individual staining of the boxed areas are presented in the bottom panel. The lack of y-tubulin and mCherry in IBPM shows that they differ from IBperi by neither recruiting cellular aggresome markers nor unrelated cytosolic proteins. (TIF) [file ppat.1007733.s012.tif]
